# Resilience to COVID-19: Socioeconomic Disadvantage Associated With Positive Caregiver–Youth Communication and Youth Preventative Actions

**DOI:** 10.3389/fpubh.2022.734308

**Published:** 2022-02-09

**Authors:** Andrew T. Marshall, Daniel A. Hackman, Fiona C. Baker, Florence J. Breslin, Sandra A. Brown, Anthony Steven Dick, Marybel R. Gonzalez, Mathieu Guillaume, Orsolya Kiss, Krista M. Lisdahl, Connor J. McCabe, William E. Pelham, Chandni Sheth, Susan F. Tapert, Amandine Van Rinsveld, Natasha E. Wade, Elizabeth R. Sowell

**Affiliations:** ^1^The Department of Pediatrics, Children's Hospital Los Angeles, University of Southern California, Los Angeles, CA, United States; ^2^University of Southern California (USC) Suzanne Dworak-Peck School of Social Work, University of Southern California, Los Angeles, CA, United States; ^3^Center for Health Sciences, SRI International, Menlo Park, CA, United States; ^4^National Center for Wellness and Recovery, Oklahoma State University Center for Health Sciences, Tulsa, OK, United States; ^5^Department of Psychiatry, University of California, San Diego, San Diego, CA, United States; ^6^Department of Psychology, University of California, San Diego, San Diego, CA, United States; ^7^Department of Psychology, Florida International University, Miami, FL, United States; ^8^Graduate School of Education, Stanford University, Stanford, CA, United States; ^9^Department of Psychology, University of Wisconsin-Milwaukee, Milwaukee, WI, United States; ^10^Department of Psychiatry and Behavioral Sciences, University of Washington, Seattle, WA, United States; ^11^Department of Psychiatry, School of Medicine, University of Utah, Salt Lake City, UT, United States

**Keywords:** adolescence, pandemic, COVID-19, socioeconomic factors, caregivers

## Abstract

Socioeconomic disadvantage is associated with larger COVID-19 disease burdens and pandemic-related economic impacts. We utilized the longitudinal Adolescent Brain Cognitive Development Study to understand how family- and neighborhood-level socioeconomic disadvantage relate to disease burden, family communication, and preventative responses to the pandemic in over 6,000 youth-caregiver dyads. Data were collected at three timepoints (May–August 2020). Here, we show that both family- and neighborhood-level disadvantage were associated with caregivers' reports of greater family COVID-19 disease burden, less perceived exposure risk, more frequent caregiver-youth conversations about COVID-19 risk/prevention and reassurance, and greater youth preventative behaviors. Families with more socioeconomic disadvantage may be adaptively incorporating more protective strategies to reduce emotional distress and likelihood of COVID-19 infection. The results highlight the importance of caregiver-youth communication and disease-preventative practices for buffering the economic and disease burdens of COVID-19, along with policies and programs that reduce these burdens for families with socioeconomic disadvantage.

## Introduction

Like the 1918 Spanish and 2009 H1N1 influenzas (1), the SARS-CoV-2 (COVID-19) pandemic has impacted lower-income populations more heavily than higher-income populations (2, 3). For example, there is greater COVID-19 infection risk, prevalence, and disease severity in lower-income and more disadvantaged regions (i.e., neighborhoods, counties) (4–13), and state-level income inequality has been shown to be associated with COVID-19-related deaths (14). By the spring and summer of 2020, there was evidence that neighborhood disadvantage was associated with greater COVID-19 prevalence in several regions across the United States (15, 16). Further, some research also suggested that greater risks of COVID-19 infection and death are linked with lower family-level household income (17, 18). All the while, those from families and neighborhoods with more socioeconomic disadvantage are more likely to suffer unemployment or other economic shocks (19), as nearly 50% of lower-income American adults have either taken a pay cut or lost their job due to the COVID-19 pandemic, compared to 32% of higher-income individuals (20). Thus, families and communities at higher risks of suffering the health and economic consequences of the pandemic ultimately have fewer resources to manage disease outbreaks, thereby worsening and exacerbating extant economic and health disparities (1).

Previous research has shown that such hardship and risk factors do not preclude adaptive coping and resilience in these populations (21). In contrast, environmental stressors, such as the stresses of economic hardship (22), may even promote greater use of coping and resilience strategies (23–25). Accordingly, in the context of COVID-19 (26), the aforementioned intersecting economic and health burdens highlight the importance of how children and families emotionally and behaviorally respond to manage challenging circumstances and reduce risk. One example concerns the preventive actions taken to reduce COVID-19 infection risk. Sociodemographic and community risk may be associated with barriers to following public health guidelines, such as differences in job-related risk or transportation (27), even though many such behaviors (e.g., social distancing, handwashing, mask-wearing) effectively mitigate infection risk and remain central components of public health messaging and pandemic response strategies (28–30). However, it is also possible that caregiver-child interactions and their relationships may be important factors influencing how youth manage the risk and prevention of COVID-19 infection and their pandemic-related worries and concern (31). Recent theoretical models have highlighted the role of caregiver-child communication in such resilience processes (i.e., sensitive and transparent communication about COVID-19 and youth's emotional states) (32, 33), and early data suggest that caregiver-child communication about COVID-19 may be protective for mental health, although measurement of communication in earlier reports was limited and not informed by consideration of both family and community disadvantage (34). Ultimately, caregiver-child communication may thus be both a potential risk-reduction process and buffer to help children stay safe and foster resilience in the face of COVID-19-related adversities.

Past studies reporting associations between neighborhood disadvantage and COVID-19 have employed ecological analyses of associations between community characteristics and community- or census-tract level COVID-19 prevalence (or other small–area levels). However, these studies are limited in their abilities to focus on both family-specific disease burden and adaptive responses to the pandemic (8–13, 15, 16) and capture the independent roles of family and neighborhood–disadvantage, which, while correlated, have separable associations with health that may operate by different mechanisms and, thus, warrant different intervention approaches (35–39). Accordingly, we focus here on data from the Adolescent Brain Cognitive Development^SM^ Study (ABCD Study® i.e., a large, national cohort of 11,878 youth and caregiver participants across 21 metropolitan areas in the United States, hereafter “ABCD”), which uniquely allows for investigating how family-level (from caregiver and youth self-reports) and geocoded neighborhood-level predictors are associated with the unequal “costs” of the pandemic on American families (1).

Specifically, ABCD permits the integration of neighborhood-level characteristics with family and youth data through census-tract-level geocoding of participants' primary residence at baseline data collection (between 2016 and 2018). The geocoded data include the area deprivation index (ADI), a 17-variable composite metric of neighborhood disadvantage derived from the U.S. Census Bureau's American Community Survey (ACS). ABCD independently collected three waves of COVID-19-related questionnaire data from its youth participants and their caregivers relatively early in the pandemic (i.e., May–August 2020), including inquiries about disease burden, perceived risk, caregiver-child communications, pandemic-related worry, and preventative behaviors to reduce the spread of the virus.

The goal of this study was to characterize associations between neighborhood disadvantage, family (household) income, and the disease burden of COVID-19 risk and worry, along with reports on family-level interactions that may alter COVID-19 perceptions, help youth manage their worries, and foster preventative actions to reduce risk of COVID-19 infection. Given the financial and health burdens of COVID-19, we hypothesized that lower household income and greater neighborhood disadvantage would be associated with greater disease burden (i.e., family risk and exposure to COVID-19), greater perceived risk of exposure/infection, and greater COVID-19-related worry. We also hypothesized that any discrepancies in the predicted relationships between socioeconomic disadvantage and COVID-19-related risk and worry may be due to differential factors in the immediate family environment related to fostering resilience and encouraging preventative actions. To our knowledge, this is the first account of how family- and geocoded neighborhood-level disadvantage are associated with caregiver and youth responses to the pandemic within a large sociodemographically diverse cohort from a geographically heterogenous sample of U.S. communities.

## Materials and Methods

### Participants

ABCD is a 10-year longitudinal study involving 21 U.S. study sites (40). Using school-based enrollment (41), ABCD enrolled 11,878 9- and 10-year-old children from an initial 22 sites. The recruitment process and the derivation of the demographically diverse target sample has been previously described (41). The following U.S. institutions (locations) are current ABCD study sites: Children's Hospital Los Angeles (Los Angeles, CA), Florida International University (Miami, FL), Laureate Institute for Brain Research (Tulsa, OK), Medical University of South Carolina (Charleston, SC), Oregon Health & Science University (Portland, OR), SRI International (Menlo Park, CA), University of California, San Diego (San Diego, CA), University of California, Los Angeles (Los Angeles, CA), University of Colorado Boulder (Boulder, CO), University of Florida (Gainesville, FL), University of Maryland, Baltimore (Baltimore, MD), University of Michigan (Ann Arbor, MI), University of Minnesota (Minneapolis, MN), University of Pittsburgh Medical Center (Pittsburgh, PA), University of Rochester (Rochester, NY), University of Utah (Salt Lake City, UT), University of Vermont (Burlington, VT), University of Wisconsin-Milwaukee (Milwaukee, WI), Virginia Commonwealth University (Richmond, VA), Washington University in St. Louis (St. Louis, MO), and Yale University (New Haven, CT). The Icahn School of Medicine at Mt. Sinai (New York, NY) was discontinued as the 22nd ABCD data-collection site early in recruitment, with many of its participants transferring to another nearby study site.

In May 2020, ABCD began disseminating questionnaires to caregiver and youth participants to assess how COVID-19 was impacting their lives. Data from the first three questionnaires (disseminated by email on May 16–22, 2020, June 24–27, 2020, and August 4–5, 2020, via unique links from ABCD) are available through the National Institute of Mental Health Data Archive (NDA) (42), which includes data for 9,268 total participants, with 5,125 youth-caregiver dyads both completing Questionnaire (Q) 1; 5,189, Q2; and 5,011, Q3. A total of 3,286 youth-caregiver dyads completed Q1 and Q2, and 2,915 participants completed Q1–Q3.

Our analyses incorporated ABCD main-study data released in November 2020, which included baseline data for 11,878 participants, 1-year-follow-up data for 11,235 participants, and 2-year-follow-up data for 6,571 participants (43); these baseline, 1-year-follow-up, and 2-year-follow-up data were all collected before the pandemic and subsequent administration of the COVID-19 questionnaires. Centralized IRB approval was obtained from the University of California, San Diego. Study sites obtained approval from their local IRBs. For the main study, caregivers provided written informed consent; children provided written assent. Accessing the COVID-19 questionnaires (i.e., clicking on the secure link) indicated willingness to participate. Data collection and analysis complied with all ethical regulations.

### COVID-19 Questionnaire

Youth and caregiver participants were emailed the COVID-19 Questionnaire; a $5 incentive was provided for completing each questionnaire (~10–15 min to complete). The analyzed items from the questionnaires are shown in the Supplementary Table 2, with some items asked only at select timepoints. Youth questionnaires were provided in English; caregiver questionnaires, English and Spanish. Data were collected via REDCap (44, 45).

#### COVID-19 Disease Burden: Family Exposure Risk and Reported Diagnoses

COVID-19 disease burden was operationally defined as caregivers' responses to two items capturing COVID-19-related burden and exposure: “Was anyone in your household at increased risk for COVID-19 due to work in healthcare or other essential jobs (such as grocery store, factory, gig economy) or use of public transit?” (Response options: No, Yes, Don't Know; Q1–Q3) and “Number of immediate family members (same household) diagnosed with coronavirus” (0–10+; Q2).

#### Perceived Risk of COVID-19

Perceived risk was operationally defined as caregivers' responses to four items (5-point Likert scale: Strongly Disagree, Disagree, Neither Disagree or Agree, Agree, Strongly Agree; Q1 and Q3): “I think it is likely that I will get coronavirus,” “I think it is likely I will be hospitalized or die from the coronavirus,” “I think it is likely that someone very close to me will get coronavirus,” and “I think it is likely that someone very close to me will be hospitalized or die from the coronavirus”. Thus, while disease burden more closely proxies actual risk (or changes in risk) given greater chance of exposure in specific situations (i.e., job, public transit), perceived risk more closely proxies subjective feelings of being at risk for exposure (i.e., relative likelihoods), without specification to actually being exposed.

#### Youth and Caregiver Worry

In Q1–Q3, both youth and caregiver participants self-reported how worried they had been about COVID-19 in the past week (5-point Likert scale: Not at All, Slightly, Moderately, Very, Extremely). Additionally, caregiver participants reported on their own perceptions of their children's worry levels about the health and non-health related consequences of COVID-19 (5-point Likert scale: Strongly Disagree, Disagree, Neither Disagree or Agree, Agree, Strongly Agree; Q1 and Q3): “My child seems worried about becoming ill or that others they know will become ill with coronavirus,” and “My child seems worried about non-health related consequences of coronavirus (e.g., financial).”

#### Caregiver-Youth Communication About COVID-19 Risk and Prevention

Caregivers were asked how often in the past week (5-point Likert scale: Never, Rarely, Occasionally, Frequently, Very Frequently; Q1-Q3) they talked with their child about (1) “the importance of handwashing for preventing the spread of germs,” (2) “the importance of social distancing,” (3) “cancellation of school and other events,” (4) “avoiding visiting friends or family,” (5) “the symptoms of coronavirus,” and (6) “protecting the elderly or other vulnerable people.” In Q3, “the importance of wearing a mask” was added. When specified, these responses (except for “wearing a mask,” as it was only asked at Q3) were averaged, for the purpose of simplicity.

#### Caregiver Support and Transparency

Caregiver support was operationally defined as the extent to which caregivers agreed (or disagreed) with two items (5-point Likert scale: Strongly Disagree, Disagree, Neither Disagree or Agree, Agree, Strongly Agree; Q1 and Q3): “I have told my child that everything will be okay,” and “I have encouraged my child not to focus on coronavirus or its impacts on people and the world.” These items are referred to as *Caregiver Reassurance* and *Caregiver Encouragement*, respectively. Caregiver transparency was operationally defined by caregivers' agreement with four statements (5-point Likert scale: Strongly Disagree, Disagree, Neither Disagree or Agree, Agree, Strongly Agree; Q1 and Q3): “I discussed with my child my own feelings about coronavirus and its impact on people and the world,” “I have avoided talking to my child about coronavirus,” “I have expressed concern to my child that they might not be fully safe from coronavirus,” and “I have prepared my child for our lives to change significantly.” For analysis, these caregiver transparency items were analyzed separately.

#### Youths' Preventative Actions

Youth participants were asked about their frequency of engaging in preventative behaviors (4-point Likert scale: “I have not done this in the last week,” “I did this some of the time last week,” I did this most of the time last week,” “I did this all the time last week”; Q1 and Q3): (1) “I stay away from people (other than those who live in my house),” (2) “I wash my hands at times other than just after I use the bathroom or before eating,” (3) “I wear a mask over my face or protective gear (e.g., gloves, things to cover my clothes),” (4) “I use Purell/other hand sanitizer,” (5) “I use Clorox/cleaners to wipe down surfaces,” (6) “I avoid touching things (e.g., phone, doorknobs),” (7) “I avoid touching people (e.g., hugging, shaking hands),” and (8) “I stay away from people inside my house (e.g., stay in another room or a certain distance away).” For analysis, given our interest in the general frequency of preventative behaviors in youth, these Likert scale data were averaged across items.

### ABCD Main-Study Data

Analyses incorporated family demographics and residential history data collected as part of the primary ABCD Study (Supplementary Table 1).

#### Socioeconomic Disadvantage

The area deprivation index (ADI) for youth participants' primary residential address at the baseline visit is a composite weighted-sum metric of neighborhood disadvantage (e.g., poverty rates, unemployment, median family income, low education; see Supplementary Table 1) (46, 47). Census-tract-level ADI, based on the 2011–2015 five-year ACS estimates (i.e., the data included and released with the ABCD dataset), was computed based on coefficient values from Kind, Jencks (46) and discretized into national percentiles for the ABCD data release (48). Scripts for computing and merging ADI (and its national percentile) with ABCD data are available: https://github.com/ABCD-STUDY/geocoding/blob/master/Gen_data_proc.R. While statistical analyses incorporated the national percentile ADI data, these data were collapsed across continuous deciles for graphing.

Caregiver-reported annual household income (before taxes, including all wages and benefits) was a continuous, ordinal factor with 10 levels (1 = < $5,000; 2 = $5,000–$11,999; 3 = $12,000–$15,999; 4 = $16,000–$24,999; 5 = $25,000–$34,999; 6 = $35,000–$49,999; 7 = $50,000–$74,999; 8 = $75,000–$99,999; 9 = $100,000–$199,999; 10 = ≥$200,000). For income, the data used in analyses were those of the most recent, non-missing data available for that participant for each of these options (i.e., as ABCD is a 10-year longitudinal study with annual visits, these variables were collected at each annual visit; however, the multiyear recruitment period of ABCD means that those annual visits were staggered across participants, such that, e.g., one participant's first and second annual visits may have occurred in 2017 and 2018, while another's may have occurred in 2018 and 2019).

#### Additional Demographic Variables

Youth participants' ages and sex at birth were available in the NDA release of ABCD's COVID-19 questionnaire data (42). Children's and caregiver's race and ethnicity were categorical factors derived from caregiver reports at baseline data collection. Race had 6 levels: “White,” “Black,” “Asian,” “American Indian or Alaska Native,” “Native Hawaiian or Other Pacific Islander,” or “Other” (e.g., multiracial). Ethnicity had two levels: “Hispanic/Latino/Latina” or “Not Hispanic/Latino/Latina.” Maximum caregiver education was a continuous, ordinal factor with 5 levels (1 = ≤12th grade, no diploma; 2 = high-school graduate, GED or equivalent; 3 = Some college with no degree, Associate's degree; 4 = bachelor's degree; 5 = master's degree, professional degree, or doctorate). As with the income data, the education data used in analyses were the most recent, non-missing education data available for that participant.

### Statistical Analyses

Integration of COVID-19 questionnaire and ABCD main-study data resulted in 21,646 data points across 9,268 participants. Participants' data were excluded listwise if the primary residential address was invalid (remaining *n* = 20,483), if the ADI score was missing or invalid (weighted sum = 0) (remaining *n* = 20,079), if there were missing data for household income, sex, age, caregiver education, race, ethnicity (remaining *n* = 19,012), or if any of a participant's questionnaires were returned out of order (e.g., Q1 was completed after Q2 was completed) (remaining *n* = 18,731). Individual questionnaire data were excluded for a participant if that questionnaire was returned after the dissemination date of the subsequent questionnaire (remaining *n* = 18,476). (Q3 responses were excluded if they were returned after October 8, 2020, the dissemination date of the fourth COVID-19 questionnaire.) While ABCD includes siblings, issues of convergence of random-effects structures led us to include only one sibling per multiparticipant family. For families in which siblings had completed different numbers of COVID-19 questionnaires (i.e., 16.4% of the families with siblings), the sibling with the most questionnaires completed was included. If multiple siblings had completed the same number of questionnaires, then the sibling included in analyses was randomly selected using MATLAB's *datasample* function (seed=1). Omnibus analyses included 16,017 data points (i.e., questionnaire responses) across 6,874 participants (Note that “participants” refers to at least one member of caregiver-child dyad, as some caregivers but not their children returned the questionnaires at each timepoint, and vice versa).

Analyses employed MATLAB's Statistics and Machine Learning Toolbox 11.7 (R2020a; MathWorks). Model output and model-fit characteristics are shown in the Supplementary Tables 4–35. Main-text statistical reporting is in the form of *t*-statistics. Effect sizes of main associations for continuous factors are represented by partial correlation coefficients (*r*_*p*_), which control for all other variables in the model and are calculated using the corresponding *t*-statistic and degrees of freedom (49); for uniformity, effect sizes were similarly calculated for generalized linear mixed-effects models. Likert-type response data were analyzed with general linear mixed-effects models (with a random initial value for iterative optimization). Count data were analyzed with generalized linear mixed-effects models assuming a Poisson distribution and a log link function. Questionnaire data with yes-no responses (excluding “Do Not Know” responses) were analyzed with generalized linear mixed-effects models assuming a binomial distribution and a logit link function (0 = no, 1 = yes). For each analysis, missing data (or missing-like data, e.g., “Do Not Know” responses) were excluded on a pairwise basis. Categorical factors were effects-coded to facilitate interpretation of main effects; continuous factors were centered to make parameter estimates more interpretable (50). Bivariate Spearman correlational analyses were conducted when specified.

Mixed-effects analyses incorporated fixed effects of ADI, maximum caregiver education, questionnaire number (centered for analysis), household income, race, and ethnicity. Analyses of data from caregiver-completed questionnaires included caregiver race and ethnicity; those from youth-completed questionnaires, youth race and ethnicity. Analyses of caregiver-reported youth worry levels included youth race and ethnicity. The fixed-effects structure of analyses of youth questionnaire data (or caregiver questionnaire data directly about their child, e.g., support, transparency, communication, worry) also included youth sex and age. After having conducted preliminary analyses of these data, quadratic terms for maximum caregiver education, household income, ADI, and questionnaire number were also included as fixed effects (Questionnaire number was not included as a fixed effect in analyses with only a single time point, and a quadratic term for questionnaire number was not included when there were only two time points). Random-effects structures included random intercepts for participant ID (for analyses of questionnaire items with repeated observations) and study site; here, because ADI referred to that of participants' residences at baseline, the study site for each participant was also that from baseline data collection (i.e., some participants were transferred from their baseline site to other sites over the first 4 years as a function of family relocation).

## Results

### Demographics and Analysis

Analyses included 16,017 observations across 6,874 unique participants (Table 1). Incomplete youth-caregiver dyad data (e.g., caregivers but not their children returning Q1 or vice versa) was not an exclusionary criterion. Relative to the entire ABCD cohort, our surveyed sample was more likely to (1) have higher incomes, (2) live in less disadvantaged census tracts, (3) identify the youth's race as white, and (4) identify the youth's ethnicity as non-hispanic (Table 1). Here, 0.4% of caregivers identified themselves as American Indian or Alaska Native, 4.1% as Asian, 11.7% as Black, 0.1% as Native Hawaiian or Other Pacific Islander, and 75.2% as white (Other, 8.5%); 14.8% of caregivers identified themselves as Hispanic/Latino/Latina. Further, ADI covaried with household income, Spearman's rho (ρ) = −0.50, *p* < 0.001. At Q1, youth participants were ~12.5 years old (range: 10.6–14.6).

**Table 1:** Demographics for the Adolescent Brain Cognitive Development (ABCD) Study.

	**Release 3.0 (%)**	**Sample in this**
	**[baseline]**	**report (%)**
**Youth sex**		
Male	6,196 (52.1%)	3,612 (52.5%)
Female	5,682 (47.8%)	3,262 (47.5%)
**Annual household income**		
< $5,000	417 (3.5%)	168 (2.4%)
$5,000–$11,999	421 (3.5%)	187 (2.7%)
$12,000–$15,999	274 (2.3%)	137 (2.0%)
$16,000–$24,999	524 (4.4%)	254 (3.7%)
$25,000–$34,999	654 (5.5%)	342 (5.0%)
$35,000–$49,999	934 (7.9%)	503 (7.3%)
$50,000–$74,999	1,499 (12.6%)	926 (13.5%)
$75,000–$99,999	1,572 (13.2%)	983 (14.3%)
$100,000–$199,999	3,315 (27.9%)	2,371 (34.5%)
≥$200,000	1,250 (10.5%)	1,003 (14.6%)
Missing/Undefined	1,018 (8.6%)	0 (0.0%)
**Area deprivation index**		
≤33 percentile (Low)	5,392 (45.4%)	3,655 (53.2%)
34–66 percentile (Mid)	3,499 (29.5%)	2,162 (31.5%)
≥67 percentile (High)	2,055 (17.3%)	1,057 (15.4%)
Missing/Undefined	932 (7.8%)	0 (0.0%)
**Youth Race**		
American Indian/Alaska Native	62 (0.5%)	26 (0.4%)
Asian	276 (2.3%)	192 (2.8%)
Black	1,869 (15.7%)	822 (12.0%)
Native Hawaiian/Pacific Islander	16 (0.1%)	8 (0.1%)
Other	1,959 (16.5%)	1,096 (15.9%)
White	7,525 (61.1%)	4,730 (68.8%)
Missing/Undefined	171 (1.4%)	0 (0.0%)
**Youth ethnicity**		
Hispanic	2,411 (20.3%)	1,258 (18.3%)
Not hispanic	9,314 (78.4%)	5,616 (81.7%)
Missing/Undefined	153 (1.3%)	0 (0.0%)
Total	11,878 (100%)	6,874 (100%)

*The “Other” Race category includes those who identified “Other Race” (n = 392) and those who identified more than one race (i.e., multiracial). As race was not the primary focus on this paper, our analyses treated race in accordance with the National Institutes of Health's Office of Management and Budget standards (https://orwh.od.nih.gov/toolkit/other-relevant-federal-policies/OMB-standards)*.

Below, we first describe associations of household income and ADI with COVID-19 disease burden, followed by evaluation of relationships between these socioeconomic variables and perceived risk of participants' themselves or someone close to them contracting or dying from COVID-19. Specifically, the primary differences between these constructs, respectively, were (1) that they captured more objective (or actual) risk vs. subjective perceptions of risk, respectively, and (2) that they reflect specific circumstances of changes in relative risk vs. perceived likelihoods of contracting/dying from COVID-19 [Here, while both education and household income are proxies of socioeconomic status, we were specifically interested in household income, as it tends to more closely approximate resource availability (51); thus, we focused on these relationships (along with ADI's) with COVID-19-questionnaire data, but statistical results for education levels are available in the Supplementary Materials]. Next, we describe socioeconomic associations with how worried caregivers and youth are about COVID-19, with caregivers also being asked whether their children are more worried about the health vs. non-health consequences of the pandemic, thereby reflecting a distinct construct from perceived likelihood of contracting/dying from COVID-19. Finally, we address associations of household income and ADI with how families are responding to the COVID-19 pandemic, in terms of caregiver-youth communication, caregiver transparency (i.e., openness about the COVID-19 pandemic), caregiver reassurance, and how youth are engaging in preventative behaviors against COVID-19. Throughout, we have identified the questionnaires (Q1, Q2, Q3) to which the data correspond (i.e., either Q1–Q3, Q1 and Q3, Q2, or Q3).

### COVID-19 Disease Burden: Family Exposure Risk and Reported Diagnoses

**Figure 1:**
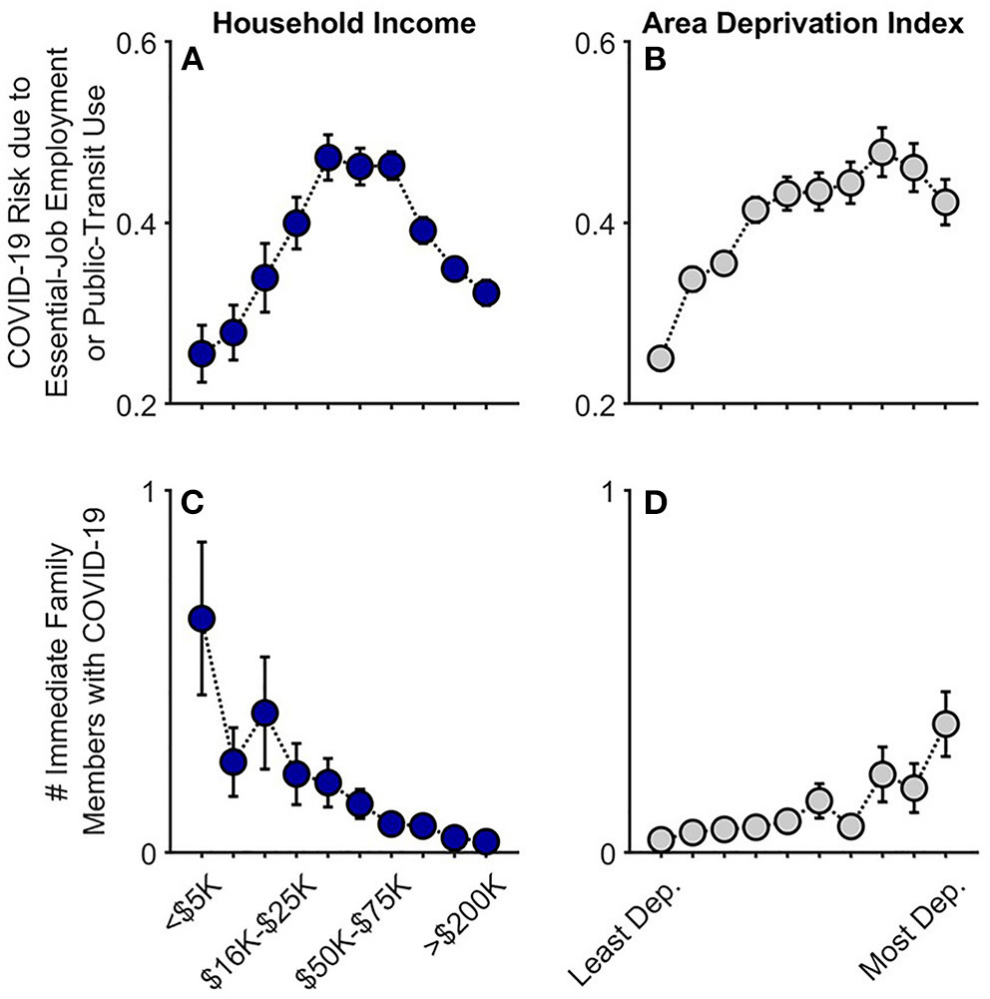
COVID-19 exposure and within-family diagnoses as functions of annual household income and their home census tract's area deprivation index. Caregiver-reported data are shown for whether individuals in participants' households were at an increased risk given job type or public-transit use **(A,B)** and the number of participants' immediate family members who had been diagnosed with COVID-19 **(C,D)**. Error bars are ±1 between-subjects standard error of the means. Analyses controlled for caregiver education, caregiver race, caregiver ethnicity, and participants' baseline study site. Given multiple observations, the job/transit risk analysis also controlled for questionnaire number and participant. Area deprivation index was collapsed across continuous deciles for graphing. Dep., Deprived.

Risk of COVID-19 exposure due to essential-job employment or public-transit use (Q1–Q3) was positively associated with ADI (i.e., greater neighborhood disadvantage), *t*_(15,313)_ = 10.45, *p* < 0.001, partial correlation coefficient (*r*_*p*_) = 0.084, but this pattern plateaued across the highest ADI tracts [(ADI)^2^], *t*_(15,313)_ = −5.48, *p* < 0.001 (Figures 1A,B; Supplementary Table 4). While risk of exposure was positively associated with household income, *t*_(15,313)_ = 9.14, *p* < 0.001, *r*_*p*_ = 0.074, there was a substantial decrease across the largest household incomes [(household income)^2^], *t*_(15,313)_ = −6.07, *p* < 0.001, in that, for household income, households with intermediate income were those more likely at increased risk of exposure.

At Q2 (late June–July 2020), 3.4% of caregivers (178/5,223) reported that at least one immediate family member (i.e., same household) had been diagnosed with COVID-19. Families with lower household incomes, *t*_(5,210)_ = −5.52, *p* < 0.001, *r*_*p*_ = 0.076, and those living in higher ADI census tracts, *t*_(5,210)_ = 3.74, *p* < 0.001, *r*_*p*_ = 0.052, reported more family members having been diagnosed with COVID-19 (Figures 1C,D; Supplementary Table 5; also see Supplementary Table 3 for a detailed breakdown of these data). In the highest ADI decile (most disadvantaged neighborhoods), 10.1% of families reported having at least one family member diagnosed with COVID-19; in the most affluent neighborhoods, 2.7%. Similarly, while 12.5% of the lowest-income households reported that at least one family member had been diagnosed with COVID-19, 2.5% of the households with the highest incomes reported at least one COVID-19 diagnosis; for families with the lowest household incomes who also lived in the most disadvantaged neighborhoods, 17.6%. Thus, as predicted here and consistent with previous reports (15–17), lower household income and residence in greater ADI census tracts were associated with greater familial COVID-19 disease burden for caregivers and youth.

### Perceived Risk

**Figure 2:**
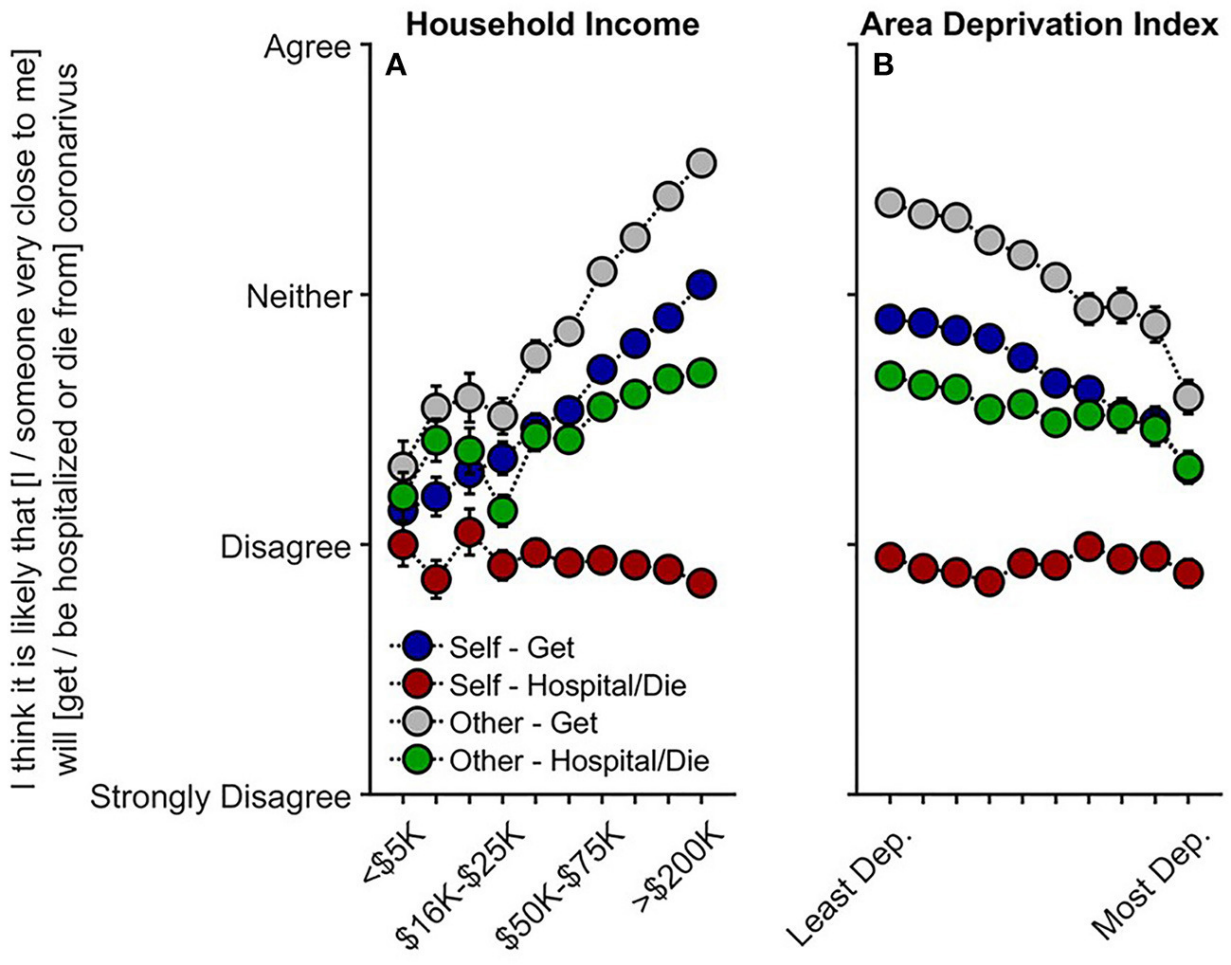
Caregivers' perceived risk of their (or someone close to them) getting and being hospitalized/dying from COVID-19 as functions of **(A)** annual household income and **(B)** their home census tract's area deprivation index. With respect to questionnaire item wording, “Self” refers to “I,” and “Other” refers to “someone very close to me.” Error bars are ±1 between-subjects standard error of the means. Analyses controlled for caregiver education, caregiver race, caregiver ethnicity, questionnaire number, participants' baseline study site, and participant. Area deprivation index was collapsed across continuous deciles for graphing. Dep., Deprived.

Contrary to our predictions, participants with lower household incomes were less likely to believe that they themselves would get COVID-19, *t*_(10,081)_ = 5.42, *p* < 0.001, *r*_*p*_ = 0.054, that someone close to them would get COVID-19, *t*_(10,082)_ = 7.10, *p* < 0.001, *r*_*p*_ = 0.071, and that someone close to them would be hospitalized or die from COVID-19, *t*_(10,082)_ = 2.96, *p* = 0.003, *r*_*p*_ = 0.030 (Q1 and Q3) (Figure 2A, Supplementary Tables 6–9). Associations between ADI and perceived risk were considerably weaker than between household income and perceived risk (Supplementary Tables 6–9). Nonetheless, similar to findings with household income, greater neighborhood disadvantage (higher ADI; Figure 2B) was associated with participants being less likely to believe they would get COVID-19, *t*_(10,081)_ = −2.57, *p* = 0.010, *r*_*p*_ = −0.026, and that someone close to them would get COVID-19, *t*_(10,082)_ = −2.07, *p* = 0.038, *r*_*p*_ = −0.021; the other perceived-risk relationships with ADI were not significant, *p*s ≥ 0.491.

As higher ADI and lower income were associated with having one or more family members diagnosed with COVID-19, we conducted sensitivity analyses including only those who had *not* had immediate family members diagnosed with COVID-19 to examine the possibility that perceived risk may differ based on experiencing positive COVID-19 tests within the household. The relationships with household income were maintained (albeit weaker) for thinking that one's self, *t*_(7,992)_ = 4.22, *p* < 0.001, *r*_*p*_ = 0.047, or someone close to him/her/them would get COVID-19, *t*_(7,993)_ = 5.59, *p* < 0.001, *r*_*p*_ = 0.062, and for whether someone close to him/her/them would be hospitalized and/or die from COVID-19, *t*_(7,993)_ = 2.10, *p* = 0.036, *r*_*p*_ = 0.023 (Supplementary Tables 10–13). However, upon accounting for reported rates of diagnosis, ADI was no longer associated with perceived risk of exposure, *p*s ≥ 0.092, suggesting that perceived risk may not align with actual disease burden or likelihood of infection.

### COVID-Related Worry

**Figure 3:**
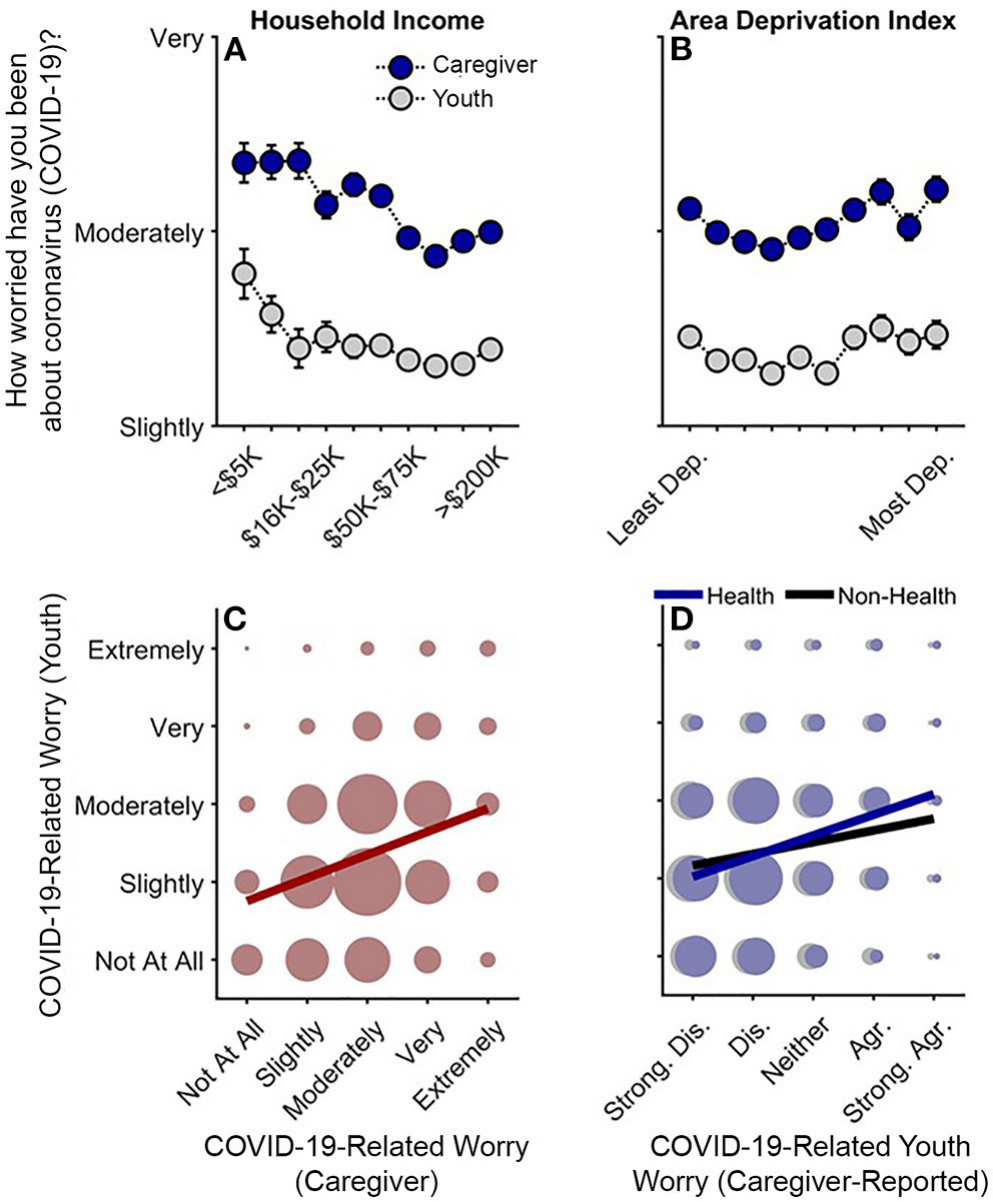
Caregiver and youth worry levels about COVID-19. **(A,B)** Worry levels as functions of annual household income and area deprivation index. Error bars are ±1 between-subjects standard error of the means. Analyses of caregiver worry controlled for caregiver education, caregiver race, caregiver ethnicity, questionnaire number, participants' baseline study site, and participant ID. Analyses of youth worry controlled for caregiver education, child race, child ethnicity, child sex, child age, questionnaire number, participants' baseline study site, and participant. Area deprivation index was collapsed across continuous deciles for graphing. **(C)** Youths' worry levels by caregivers' worry levels. **(D)** Youths' worry levels by caregiver-reported youth worry levels about the health- and non-health-related consequences of the COVID-19 pandemic. **(C,D)** Circle size reflects the number of datapoints at each x-y coordinate. The solid lines are best fitting simple regression lines. Dep., Deprived; Strong. Dis., Strongly disagree; Dis., Disagree; Agr., Agree; Strong. Agr., Strongly agree.

Lower household income was related to greater caregiver worry (Q1–Q3), *t*_(15,357)_ = −2.87, *p* = 0.004, *r*_*p*_ = −0.023. Caregiver worry was also positively associated with ADI in higher ADI tracts [(ADI)^2^], *t*_(15,357)_ = 2.05, *p* = 0.041] (Figures 3A,B; Supplementary Table 14). Although youth self-reported worry was inversely associated with household income (Q1-Q3), *t*_(12,510)_ = −2.32, *p* = 0.020, *r*_*p*_ = −0.021, with this association plateauing at greater income levels [(household income)^2^], *t*_(12,510)_ = 2.90, *p* = 0.004, youth self-reported worry was neither linearly nor quadratically related to ADI, *p*s ≥ 0.072 (Supplementary Table 15). Caregiver-reported youth worry levels about the health- and non-health-related consequences (e.g., financial) of COVID-19 were also negatively associated with household income, *p*s <0.001, but not ADI, *p*s ≥ 0.342 (Q1 and Q3) (Supplementary Tables 16, 17). Analyses also indicated that greater disease burden was related to greater caregiver but not youth worry levels (see Supplementary Material, “*COVID-19-Related Worry and Disease Burden*”).

Youths' self-reported worry levels were highly correlated with, but noticeably lower than, their caregivers' worry levels, Spearman's rho (ρ) = 0.28, *p* < 0.001 (Figure 3C) (Caregiver: *M* = 3.01, *SEM* = 0.01; Youth: *M* = 2.36, *SEM* = 0.01). While youth were only asked about general COVID-19-related worry, youth's self-reported worry was more highly correlated with their caregivers' report on their health-related, ρ = 0.26, *p* < 0.001, than non-health-related worry, ρ = 0.14, *p* < 0.001 (Figure 3D), suggesting that youth's general self-reported COVID-19-related worry was more related to their concerns about getting sick from COVID-19 rather than its non-health-related consequences.

### Families' Responses to the COVID-19 Pandemic

Lower household income was associated with both greater disease burden (risk/exposure) and greater youth and caregiver worry, while higher ADI was associated with greater disease burden. However, greater socioeconomic disadvantage across both levels was associated with less perceived risk. To determine whether families with more socioeconomic disadvantage were differentially engaging in potential coping or disease-risk reduction strategies given greater COVID-19 risk and disease burden, we analyzed indicators of caregiver-youth communication about COVID-19 risk and prevention, caregiver reassurance and transparency, and youth's COVID-19 preventative behaviors.

#### Caregiver-Youth Communication About COVID-19 Risk and Prevention

**Figure 4:**
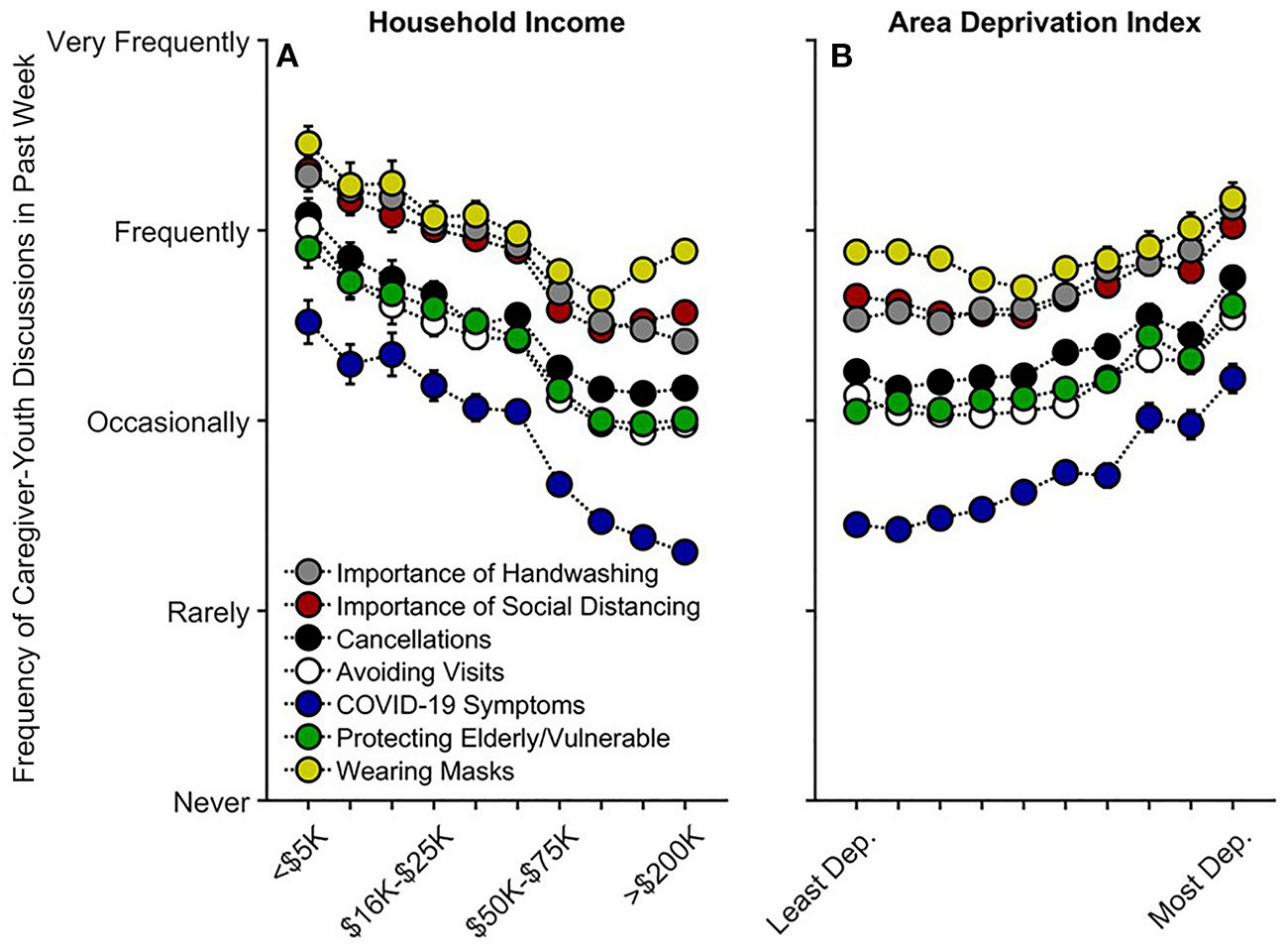
Caregiver participants' communication frequency with youth on factors related to COVID-19 risk and prevention as functions of **(A)** annual household income and **(B)** their home census tract's area deprivation index. Error bars are ±1 between-subjects standard error of the means. Analyses controlled for caregiver education, caregiver race, caregiver ethnicity, child sex, child age, questionnaire number, participants' baseline study site, and participant, except for “Wearing Masks,” the analysis for which did not include questionnaire number or participant ID due to its only having one timepoint. Area deprivation index was collapsed across continuous deciles for graphing. Dep., Deprived.

Lower household income was associated with more communication on all topics queried regarding COVID-19 prevention (Q1–Q3) (Figure 4A): the importance of handwashing, *t*_(15,063)_ = −3.12, *p* = 0.002, *r*_*p*_ = −0.025; the importance of social distancing, *t*_(15,062)_ = −5.03, *p* < 0.001, *r*_*p*_ = −0.041; cancellations of school and other events, *t*_(15,061)_ = −5.27, *p* < 0.001, *r*_*p*_ = −0.043; avoiding visits with friends/family, *t*_(15,061)_ = −7.11, *p* < 0.001, *r*_*p*_ = −0.058; COVID-19 symptoms, *t*_(15,057)_ = −6.08, *p* < 0.001, *r*_*p*_ = −0.049; protecting the elderly/vulnerable, *t*_(15,061)_ = −4.55, *p* < 0.001, *r*_*p*_ = −0.037; and, wearing masks, *t*_(4,804)_ = −3.34, *p* = 0.001, *r*_*p*_ = −0.048 (Supplementary Tables 22–28; “wearing masks” was only queried at Q3). Thus, families with lower vs. higher incomes were speaking with their children about COVID-19 prevention. Aside from caregivers' talking about COVID-19 symptoms and handwashing, *p*s ≥ 0.359, these associations tended to plateau at the highest income levels [(household income)^2^], *p*s ≤ 0.028.

For ADI, while there were small negative associations between ADI and frequency of caregiver-youth discussions on three queried COVID-19 prevention topics [importance of social distancing, *t*_(15,062)_ = −2.81, *p* = 0.005, *r*_*p*_ = −0.023; avoiding visits with friends/family, *t*_(1,561)_ = −3.06, *p* = 0.002, *r*_*p*_ = −0.025; and, wearing masks, *t*_(4,804)_ = −2.34, *p* = 0.019, *r*_*p*_ = −0.034], there were significant positive quadratic terms for ADI for each of these topics, *p*s ≤ 0.016 (Figure 4B, Supplementary Tables 22–28). To better understand the quadratic relationships between ADI and caregiver-youth communication on COVID-19 prevention, we conducted bivariate ADI-by-caregiver/youth-communication correlational probe analyses (for all prevention topics) separately for those with ADI ≤ 40th percentile (Low ADI; *n* = 4,414 participants) and for those with ADI > 40th percentile (High ADI; *n* = 2,460 participants), given the minimal change in caregiver-youth communication below the 40th percentile (i.e., the 40% least deprived per national percentile; Figure 4B). For High ADI participants, there were significant positive correlations between ADI and caregiver-youth communication frequency on all queried topics related to COVID-19 risk/prevention, ρs ≥ 0.12, *p*s <0.001. In contrast, these relationships were substantially weaker for Low ADI participants (hand washing: ρ = 0.01, *p* = 0.484; social distancing: ρ = −0.04, *p* < 0.001; cancellations: ρ = −0.01, *p* = 0.338; avoiding visits: ρ = −0.04, *p* < 0.001; COVID-19 symptoms: ρ = 0.02, *p* = 0.041; protecting the elderly/vulnerable: ρ = 0.01, *p* = 0.252; wearing masks: ρ = −0.04, *p* = 0.018), further suggesting that caregivers in more disadvantaged neighborhoods were talking more with their children about COVID-19 prevention (Figure 4).

**Figure 5:**
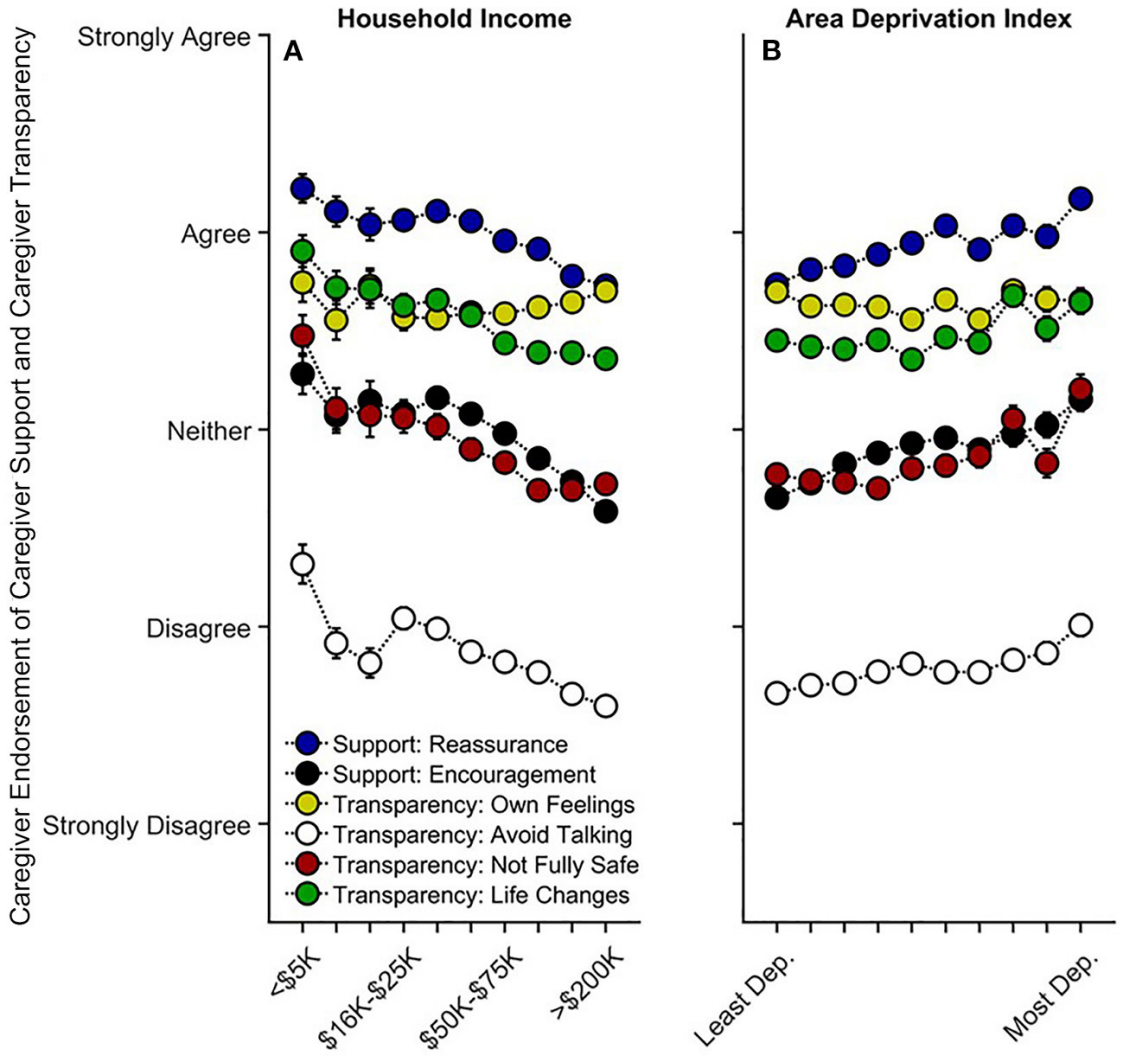
Caregiver support and transparency as functions of **(A)** annual household income and **(B)** their home census tract's area deprivation index. Error bars are ±1 between-subjects standard error of the means. Analyses controlled for caregiver education, caregiver race, caregiver ethnicity, child sex, child age, questionnaire number, participants' baseline study site, and participant. Caregiver “reassurance” refers to how much caregivers agreed with, “I have told my child that everything will be okay.” Caregiver encouragement refers to how much caregivers agreed with, “I have encouraged my child not to focus on coronavirus or its impacts on people and the world.” “Own Feelings” refers to how much caregivers agreed with “I discussed with my child my own feelings about coronavirus and its impact on people and the world.” “Avoid Talking” refers to how much caregivers agreed with, “I have avoided talking to my child about coronavirus.” “Not Fully Safe” refers to how much caregivers agreed with, “I have expressed concern to my child that they might not be fully safe from coronavirus.” “Life Changes” refers to how much caregivers agree with, “I have prepared my child for our lives to change significantly.” Area deprivation index was collapsed across continuous deciles for graphing. Dep., Deprived.

Lower household income was also associated with more caregiver encouragement, *t*_(9,839)_ = −2.72, *p* = 0.007, *r*_*p*_ = −0.027, but there was no relationship between income and caregiver reassurance, *t*_(9,842)_ = −1.18, *p* = 0.239, *r*_*p*_ = −0.012 (Q1 and Q3) (Supplementary Tables 29, 30). Except for talking about their own COVID-19-related feelings, *p* = 0.698, caregivers with lower household incomes were more likely to avoid talking to their child about COVID-19, *t*_(9,842)_ = −5.09, *p* < 0.001, *r*_*p*_ = −0.051, more likely to tell their child that they may not be fully safe from COVID-19, *t*_(9,841)_ = −4.40, *p* < 0.001, *r*_*p*_ = −0.044, and more likely to prepare their child that their lives may change significantly, *t*_(9,842)_ = −4.28, *p* < 0.001, *r*_*p*_ = −0.043 (Q1 and Q3) (Figure 5A, Supplementary Tables 31–34). Higher ADI (greater neighborhood disadvantage) was associated with more caregiver reassurance, *t*_(9,842)_ = 2.58, *p* = 0.010, *r*_*p*_ = 0.026, and encouragement, *t*_(9,839)_ = 2.43, *p* = 0.015, *r*_*p*_ = 0.024 (Figure 5B, Supplementary Tables 29, 30). However, there were no linear or quadratic associations with ADI and caregiver transparency items, *p*s ≥ 0.300 (Figure 5B, Supplementary Tables 31–34). Thus, like caregiver-youth communication on COVID-19 risk/prevention, caregivers with lower household incomes and/or living in higher ADI tracts may be providing their child with greater preventative and anxiety-reducing emotional support in the wake of increased risk of COVID-19 exposure. Greater frequency of caregiver-youth discussions on COVID-19 prevention was also associated with less perceived risk, particularly in High ADI participants (see Supplementary Material, “*Caregiver-Youth Communication and Perceived Risk*”).

#### Youths' Preventative Actions

**Figure 6:**
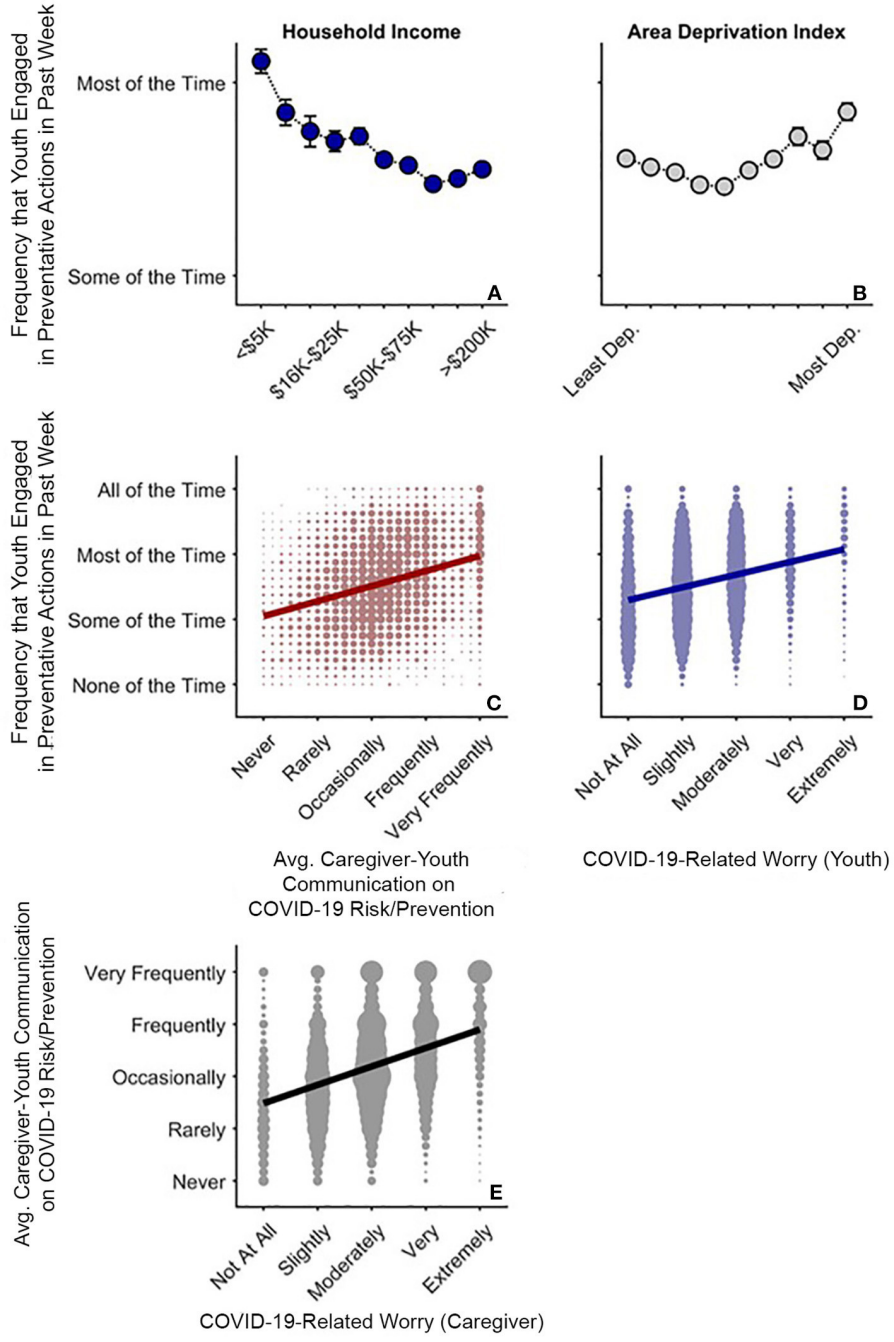
COVID-19 risk and prevention as functions of COVID-19-related worry in caregivers and youth. **(A,B)** Average (Avg.) frequency that youth endorsed COVID-19-related preventative behaviors as functions of annual household income and area deprivation index. Error bars are ±1 between-subjects standard error of the means. Analysis controlled for caregiver education, child race, ethnicity, sex, and age, questionnaire number, participants' baseline study site, and participant. Area deprivation index was collapsed across continuous deciles for graphing. **(C)** Frequency of youths' preventative behaviors by caregiver-youth risk/prevention communication frequency (i.e., averaged data from Figure 4). **(D)** Frequency of youths' preventative behaviors by youth COVID-19-related worry. **(E)** Frequency of caregiver-child risk/prevention communication by caregiver COVID-19-related worry. **(C–E)** Circle size reflects the number of datapoints at each x-y coordinate. Solid lines are best fitting simple regression lines.

In the face of greater COVID-19 disease burden within families, youth of families with lower incomes and those living in more disadvantaged neighborhoods reported greater engagement in preventative actions (Q1 and Q3). Greater household income was associated with lesser frequency of youths' preventative actions, *t*_(8,071)_ = −4.05, *p* < 0.001, *r*_*p*_ = −0.045, plateauing at the greatest household incomes [(household income)^2^], *t*_(8,071)_ = 3.48, *p* = 0.001 (Figure 6A, Supplementary Table 35). More frequent youth preventative actions was also evident in the more disadvantaged neighborhoods [(ADI)^2^], *t*_(8,071)_ = 3.41, *p* = 0.001 (Figure 6B). As with caregiver-youth communication frequency, there was a strong positive relationship between ADI and youth preventative actions for High ADI participants, ρ = 0.16, *p* < 0.001, but a weaker, negative relationship for Low ADI participants, ρ = −0.07, *p* < 0.001.

The direct relationships between socioeconomic disadvantage and youths' engagement in preventative behaviors mirrored the relationships with how often caregivers reported discussing prevention with their children. This finding was confirmed via a strong association between the average frequencies of youths' preventative actions and caregiver-youth discussions on COVID-19 risk and prevention, ρ = 0.30, *p* < 0.001 (Figure 6C). While youth who were more worried about COVID-19 also engaged more in COVID-19 preventative actions, ρ = 0.28, *p* < 0.001 (Figure 6D), caregivers who were more worried about COVID-19 were also more likely to talk to their children about COVID-19 risk and prevention strategies (see Figure 3), ρ = 0.37, *p* < 0.001 (Figure 6E). More frequent caregiver-youth discussions on COVID-19 prevention and greater youth engagement in preventative behaviors were also associated with greater caregiver support and transparency (see Supplementary Material, “*Preventative Actions, Caregiver Support, and Caregiver Transparency*”). Thus, frequency of caregiver-youth communication on COVID-19 risk/prevention paralleled how often children endorsed engaging in preventative actions, both occurring more often in families with lower household incomes and/or those living in more disadvantaged census tracts.

## Discussion

To our knowledge, this is the first study describing associations between how caregivers and their children are responding to COVID-19, with respect to disease burden, perceived risk, communication, emotional distress, and behaviors to reduce its spread, in the context of family- and neighborhood-level socioeconomic disadvantage. As in previous reports (1–18), we showed greater COVID-19 disease burden in households with lower incomes and/or living in more disadvantaged neighborhoods (Figure 1). In contrast to research employing ecological analyses of the socioeconomic disparities of COVID-19's impact, our report uniquely integrated neighborhood- and family-level socioeconomic data to elucidate how multilevel socioeconomic disadvantage in a nationwide sample related to caregivers' and youths' responses to the ongoing pandemic. While worry levels were higher among families with family-level socioeconomic disadvantage (Figure 3A), supporting our hypotheses, families with greater family- and neighborhood-level disadvantage reported more caregiver-youth discussion on ways to reduce the spread of COVID-19 (Figure 4), more frequent supportive and transparent discussions about COVID-19 between caregivers and their children (Figure 5), and more frequent youth preventative actions (Figure 6). While these latter results did not support our hypotheses, they do corroborate the literature reflecting greater adaptive coping strategies in the presence of more environmental stressors (21–25). Individuals living in high-stress environments with more disadvantage may thus be more adept at coping with future crises [see (52)]. Accordingly, protective actions in these families with more socioeconomic disadvantage may have contributed to less perceived risk of COVID-19 infection (i.e., less belief of getting or being hospitalized/dying from COVID-19; Figure 2 and Supplementary Figure 1). Ultimately, separable associations with neighborhood- and family-level socioeconomic disadvantage suggest that consideration of the disproportionate impact of COVID-19, family responses, interventions, or policy approaches to reduce the corresponding inequities must consider both families and their communities (38).

Past reports have described how family- and neighborhood-level factors may heighten vulnerabilities to natural/manmade disasters and disease outbreaks (1–3), such as the 9/11 terrorist attacks (53) and Hurricane Sandy (54). The COVID-19 pandemic has been no exception. While those of higher socioeconomic status (SES) may have been exposed to COVID-19 earlier in the pandemic (7), potentially experiencing the greatest changes to their daily lives (55), the pandemic has disproportionately burdened the families with lower household incomes and/or those living in more disadvantaged regions (2, 8–13, 15–17, 56). The Centers for Disease Control's Social Vulnerability Index (SVI) (https://www.atsdr.cdc.gov/placeandhealth/svi/index.html) and related SVI metrics have also been linked to higher COVID-19 case and death rates (4–6, 8). While our analyses revealed similar patterns (i.e., greater family risk and exposure given greater socioeconomic disadvantage), our data offer unique insight into how these differential levels of disadvantage are associated with individual caregiver-youth processes and behaviors given such ecological risks. Our results suggest that caregivers and families with more socioeconomic disadvantage may be proactively taking steps to reduce disease burden, suggesting that the necessary public health and policy interventions to reduce inequitable burdens of COVID-19, and, perhaps, reduce mental health problems that emerge from the pandemic, would be strengthened by collaborating and coordinating with communities, building on their strengths to focus on prevention.

Previous research has shown that adolescents (13–18 years old) with stronger views on the severity of COVID-19 were more likely to engage in social distancing and disinfecting behavior (57). Along with research showing that greater COVID-19-related worry (58) and fear (59) were related to more behavioral change in adults, our results demonstrate that COVID-19-related worry was highly correlated with caregiver and youth engagement in behaviors related to risk-reduction and prevention (Figure 6). As disease burden was more closely aligned with COVID-19-related concern and preventative action, the reduction in perceived risk given lower household incomes and higher ADI (Figure 2) may be partially due to heightened vigilance related to the pandemic (i.e., participants may be less likely to think that they or someone close to them will get COVID-19 because they are taking more preventative action to reduce its spread; Supplementary Figure 1). Caregivers may be acting as buffers for how their children are emotionally and behaviorally responding to COVID-19, in that youths' COVID-19-related worry and response may better reflect their caregivers' worries than the state of the surrounding community (i.e., how neighborhood disadvantage is associated with COVID-19 disease burden). While children of families with lower incomes may be more cognizant of their own families' SES, they may be less affected by community risk if their caregivers adaptively incorporate strategies to reduce environmental influences of COVID-19 infection and any associated emotional distress.

During the pandemic, public transit use in Chicago and New York City declined less in more disadvantaged areas, which are home to many “essential” workers (60, 61), suggesting that those living in regions most vulnerable to COVID-19 (8, 9, 15, 16) may not have the same luxury to engage in the same COVID-19 prevention efforts as individuals with more socioeconomic advantage (7, 27, 61, 62). Papageorge et al. (62) showed that although individuals with higher incomes were more likely to engage in COVID-19-related protective behaviors, those who had experienced losses to household income did so as well. While we cannot infer causality, our data suggest that individuals with socioeconomic disadvantage may be partially counteracting such elevated vulnerability via frequent discussions with their children on COVID-19 risk and prevention actions, even despite potentially greater costs to engage in such behaviors (62). As the relationship between caregiver stress and caregiver involvement in their children's emotional regulation may be more pronounced in socioeconomically at-risk (than non-at-risk) families, with caregiver involvement being potentially more effective at reducing children's negative emotions in families who are at risk (55), it is imperative to develop strategies to support families with more socioeconomic disadvantage during (and in the aftermath of) the global crisis brought upon by the pandemic.

While it may seem contradictory that COVID-19 burden is elevated in the same populations performing more preventative behaviors, it is not possible to know how our participants would have been affected if they had *not* done those preventative behaviors (i.e., the burden may have been considerably larger). Analytically, our COVID-19-burden/perceived-risk data were also collected from caregivers, while the preventative-behavior data were collected from youth participants; relatedly, the perceived-risk data also applied to the participants' close relationships (i.e., “someone very close to me”), and we do not have data on these other individuals' behaviors. Further, since our analyses are functionally cross-sectional, we may be observing such associations because the higher risks are motivating individuals to engage in more preventive actions. Ultimately, given the socioeconomic disparities in COVID-19 burden, it is not reasonable to assume that disparities cannot exist even in the presence of higher preventive behaviors or that preventative behaviors cannot occur even in circumstances of elevated risk: When systemic inequities create different baseline risk levels or circumstances, even higher levels of prevention may reduce but not always eliminate all inequities.

Our analyses revealed many statistically significant quadratic terms, most apparent in the disease-burden analyses for household income (Figure 1). Here, households with relatively intermediate household incomes expressed the greatest COVID-19 risk given job type and public transit use, a pattern possibly been driven by both occupation type and employment (e.g., greater likelihood of working remotely in households with higher incomes). Thus, polytonic relationships between family- and neighborhood-level SES and COVID-19-related risk, behavior, and prevention should be examined to identify unique risk factors for policy intervention.

Our results offer critical insight into associations between family- and neighborhood-level disadvantage and how caregivers and their children are responding to the COVID-19 pandemic, but these are not without limitations. The observational nature of ABCD precludes inferring causality regarding ADI and household income, as well as the directionality of caregiver-youth dyadic behavior (e.g., whether COVID-19-related discussions were caregiver- or youth-initiated); as we could not definitively establish reasonable temporal order, we did not conduct causal path analyses, considering the potential for substantial biases in such cross-sectional mediational analysis (63–65). However, an emerging strength of ABCD is its longitudinal design in a large cohort, permitting, for example, continued analyses of youth development with respect to differential exposures to COVID-19. Also, while the current report uses self-reports of disease burden, preventative behaviors, etc., the established rapport with ABCD families across study sites and similar patterns for multiple phenomena across caregiver and youth reports provide confidence in the data. Given ABCD's rigorous biospecimen collection protocol (e.g., saliva, blood), ABCD has discussed incorporating COVID-19 tests and antibody testing in future protocols. This will be an advantage over self-report data, as data for total family members diagnosed with COVID-19 may be underestimations of true case rates, especially for the families with socioeconomically disadvantage who may have limited access to testing locations and vaccinations (66).

With respect to youth preventative actions, we cannot distinguish between those who did not leave their homes (and, e.g., did not need to wear a mask) vs. those who did (and, e.g., chose not to wear one). Lastly, the ADI used here was based on participants' primary residential addresses at baseline data collection of ABCD, a metric based on the 2011–2015 5-year ACS summary (i.e., the data included and released with the ABCD dataset). Further, these ABCD data only include baseline residential data, so we cannot account for relocation. However, even though the ADI data are based on data from years before the onset of the pandemic, research has shown that deprivation levels of individuals' neighborhoods are often relatively stable over time, even when participants move (67), a phenomenon that may persist across generations (68), suggesting that geocoding of addresses collected at baseline may be a sufficient proxy for participants who have moved (69). While nearly half of participants in the current sample were of families with higher incomes, our sample still encompassed the demographic diversity of the ABCD cohort with respect to including individuals living in the most disadvantaged neighborhoods with the lowest household incomes (see Table 1). It remains possible that this sample is not representative of the population, particularly for families with more socioeconomic disadvantage, further highlighting the need to prioritize research on and provide support for the families with more socioeconomic disadvantage who will carry the heaviest burdens of the COVID-19 pandemic (1–3).

In conclusion, our data suggest that families with more socioeconomic disadvantage may be promoting greater resilience in their children (or protecting them from greater COVID-19 disease burden) per more frequent discussions on COVID-19 risk/prevention, greater caregiver support, and more direct COVID-19-related conversations. Youth in more disadvantaged situations also reported greater preventative behaviors to reduce the likelihood of contracting COVID-19. While self-report COVID-19 data will continue to be invaluable to better understand how disasters impact adolescent development, contextualizing these data with respect to neighborhood factors (38) may greatly inform how community leaders, policy makers, healthcare workers, and caregivers can alleviate the economic, health, and psychological impact of such disasters. Overall, our results have critical implications for COVID-19-related physical and emotional health of children and their caregivers whilst educators and government officials continue to consider the many factors to reopen schools and businesses to full capacity (70). Along with the much-needed actions to reduce disparities and structural inequities that contribute to disease risk, it may be helpful to encourage caregiver guidance and open COVID-19-related discussions between caregivers and their children on preventative behaviors, as this may help (1) reduce socioeconomic inequalities of COVID-19 disease burden, (2) promote resilience to natural disaster in children, and (3) encourage individuals to modify their own behaviors to proactively mitigate the scourge of the next pandemic. Ultimately, while advocacy for such preventative behaviors is important, it does not supplant the dire need to alleviate the systemic inequities that produce such disparities.

As ABCD progresses, its linking of geocoded residential history data to the COVID-19 questionnaire data will provide key insight into how early and current environments are associated with the health and mental health outcomes related to COVID-19 as well as subsequent trajectories of brain, emotional, social, and cognitive development (71). We urge public officials to aid and support families with socioeconomic disadvantage beyond the actions that they are already incorporating themselves, so as to mitigate, and eventually eliminate, the persistent unequal socioeconomic and health burdens that are unveiled and exacerbated in times of crisis.

## Data Availability Statement

The datasets presented in this study can be found in online repositories. The names of the repository/repositories and accession number(s) can be found at: https://nda.nih.gov/abcd.

## Ethics Statement

The studies involving human participants were reviewed and approved by University of California, San Diego IRB. Written informed consent to participate in this study was provided by the participant's legal guardian/next of kin. Centralized IRB approval was obtained from the University of California, San Diego. Study sites obtained approval from their local IRBs. For the main study, caregivers provided written informed consent and children provided written assent. Accessing the COVID-19 questionnaires (i.e., clicking on the secure link) indicated willingness to participate. Data collection and analysis complied with all ethical regulations.

## Author Contributions

AM, DH, FCB, FJB, SB, AD, KL, ST, NW, and ES: conception and design of experiments and analysis. AM, FCB, FJB, SB, AD, MRG, MG, OK, KL, CM, WP, CS, ST, AR, NW, and ES: data collection. AM, DH, FCB, FJB, SB, AD, MRG, MG, OK, KL, CM, WP, CS, ST, AR, NW, and ES: analysis and interpretation. AM, DH, and ES: writing of the paper. All authors contributed to the article and approved the submitted version.

## Funding

This ABCD Study® was supported by the National Institutes of Health and additional federal partners under award numbers National Institutes of Health grants U01DA041048, U01DA050989, U01DA051016, U01DA041022, U01DA051018, U01DA051037, U01DA050987, U01DA041174, U01DA041106, U01DA041117, U01DA041028, U01DA041134, U01DA050988, U01DA051039, U01DA041156, U01DA041025, U01DA041120, U01DA051038, U01DA041148, U01DA041093, U01DA041089, U24DA041123, and U24DA041147. Addition support for this work was made possible from supplements to U24DA041123 and U24DA041147, the National Science Foundation grant NSF 2028680, and Children and Screens: Institute of Digital Media and Child Development Inc. A full list of supporters is available at https://abcdstudy.org/federal-partners.html.

## Author Disclaimer

This manuscript reflects the views of the authors and may not reflect the opinions or views of the NIH or ABCD consortium investigators.

## Conflict of Interest

FCB and OK are employed by SRI International, a non-profit research organization. The authors declare that this study received funding from the National Institutes of Health, the National Science Foundation, and Children and Screens: Institute of Digital Media and Child Development Inc. The funders were not involved in the study design, collection, analysis, interpretation of data, and the writing of this article or the decision to submit it for publication. All authors declare no other competing interests.

## Publisher's Note

All claims expressed in this article are solely those of the authors and do not necessarily represent those of their affiliated organizations, or those of the publisher, the editors and the reviewers. Any product that may be evaluated in this article, or claim that may be made by its manufacturer, is not guaranteed or endorsed by the publisher.
