# Supplementary material for: Resilience to COVID-19: Socioeconomic Disadvantage Associated With Positive Caregiver–Youth Communication and Youth Preventative Actions
Source: Front Public Health. 2022 Feb 9;10:734308. doi: 10.3389/fpubh.2022.734308 (PMC8865385; doi:10.3389/fpubh.2022.734308)
Supplement: Supplementary file 1 [file Data_Sheet_1.docx]

Supplementary Material

**1 Supplementary Text**

**1.1 COVID-19-Related Worry and Disease Burden**

As described in the main text, family- but not neighborhood-level disadvantage was associated with youth’s worry levels, suggesting that, while youths’ and caregivers’ COVID-19-related worry was highly correlated, youth’s worry levels may be less influenced by COVID-19 disease burden. Indeed, while caregivers’ worry was significantly greater if they reported an increased risk of COVID-19 exposure, *p* = .001, youth worry levels were not, *p* = .191 [including this factor of increased risk (disease burden) did reveal an association between ADI and youth worry levels, *t*(11880) = ‑1.97, *p* = .049, *r_p_* = ‑.018, albeit a marginally significant one] (Tables S18-S19). Similarly, caregiver-reported youth worry levels were also not significantly associated with increased risk of exposure [health-related consequences: *p* = .051; non-health-related consequences: *p* = .602] (Tables S20-S21). Youth-reported worry levels were also not related to the number of immediate-household family members diagnosed with COVID-19, ρ = .02, *p* = .193, while caregivers’ worry levels were, ρ = .07, *p* < .001.

**1.2 Caregiver-Youth Communication and Perceived Risk**

Despite the greater disease burden in families living with low household incomes and/or in high ADI tracts, these families’ actions to reduce the toll of COVID-19 may be contributing to the reduced perceived risk. In fact, while increased frequency of caregiver-youth COVID-19 risk/prevention discussion was associated with feeling less likely that the caregivers themselves, ρ = ‑.05, *p* < .001, or someone they know would get COVID-19, ρ = ‑.06, *p* < .001, these inverse relationships were considerably stronger in the High ADI participants, ρs = ‑.09, *p*s < .001 (Low ADI, Self-Get COVID-19, ρ = ‑.01, *p* = .308; Low ADI, Other-Get COVID-19, ρ = ‑.03, *p*s = .027) (Figure S1).

**1.3 Preventative Actions, Caregiver** **Support, and Caregiver** **Transparency**

Children were more likely to take COVID-19 preventative action if their caregivers (1) avoided talking to their child about COVID-19, ρ = .04, *p* = .001, (2) expressed concern over their child not being fully safe from COVID-19, ρ = .15, *p* < .001, (3) prepared their child for their lives to change significantly, ρ = .17, *p* < .001, and (4) discussed their own COVID-19-related feelings with their child, ρ = .08, *p* < .001. Except for caregivers’ avoiding talking to their child about COVID-19, ρ = .03, *p* = .002, there were highly significant positive relationships between caregiver-youth discussions on COVID-19 risk/prevention topics (average frequency) and caregiver support, ρs ≥ .07, *p*s ≤ .001, as well as with caregivers’ discussing their own COVID-19-related feelings with their child, ρ = .21, *p* < .001, expressing concern over their child not being fully safe from COVID-19, ρ = .31, *p* < .001, and preparing their child for their lives to change significantly, ρ = .32, *p* < .001.

**2 Supplementary Figures and Tables**

**2.1 Supplementary Figure**

Avg. Caregiver-Youth

Communication on

COVID-19 Risk/Prevention

Avg. Caregiver-Youth

Communication on

COVID-19 Risk/Prevention


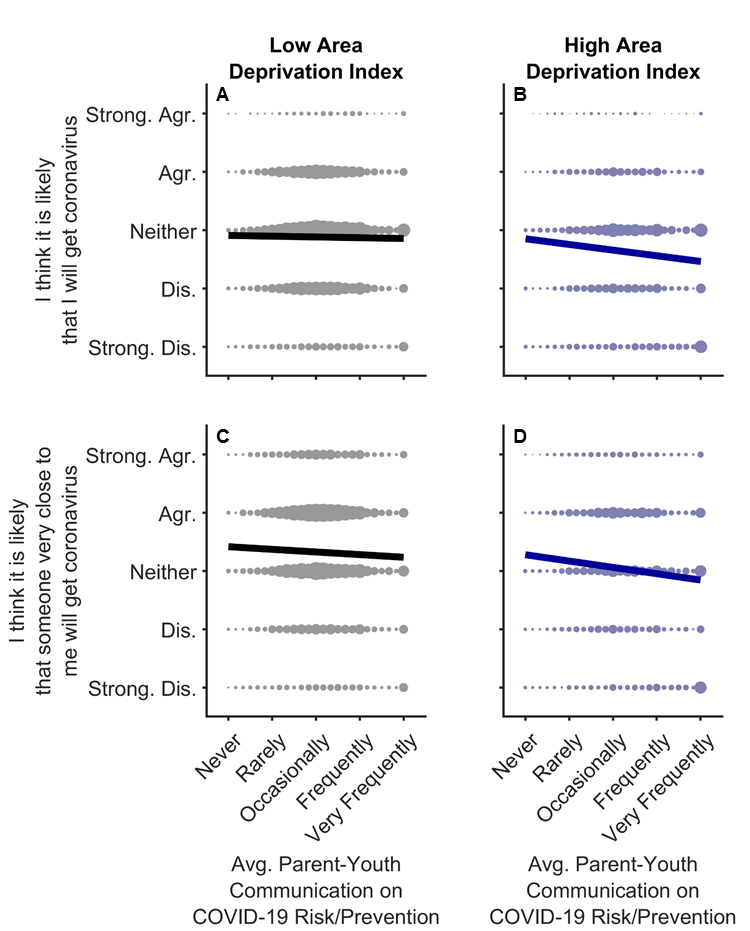


**Supplementary Figure 1.** **Perceived risk as a function of average frequency of caregiver-youth communication on COVID-19 risk/prevention, separated by high versus low area-deprivation levels**. Circle size reflects the number of datapoints at each x-y coordinate. The solid lines are best fitting simple regression lines. Strong. Dis. = Strongly Disagree. Dis. = Disagree. Agr. = Agree. Strong. Agr. = Strongly Agree.

**2.2 Supplementary Tables**

**Supplementary Table 1. NDA Data Release 3.0 Variables**

| **Data Tables and Variables (NDA Release 3.0)** | **Variable Labels in Current Report** |
| --- | --- |
| **Baseline Demographics (pdem02)** |  |
| demo_comb_income_v2 | Household Income |
| demo_prnt_ed_v2 | Caregiver 1 Education Level |
| demo_prtnr_ed_v2 | Caregiver 2 Education Level |
| demo_race_a_p___10 | Youth Race (White) |
| demo_race_a_p___11 | Youth Race (Black) |
| demo_race_a_p___12 | Youth Race (American Indian) |
| demo_race_a_p___13 | Youth Race (Alaska Native) |
| demo_race_a_p___14 | Youth Race (Native Hawaiian) |
| demo_race_a_p___15 | Youth Race (Guamanian) |
| demo_race_a_p___16 | Youth Race (Samoan) |
| demo_race_a_p___17 | Youth Race (Other Pacific Islander) |
| demo_race_a_p___18 | Youth Race (Asian Indian) |
| demo_race_a_p___19 | Youth Race (Chinese) |
| demo_race_a_p___20 | Youth Race (Filipino) |
| demo_race_a_p___21 | Youth Race (Japanese) |
| demo_race_a_p___22 | Youth Race (Korean) |
| demo_race_a_p___23 | Youth Race (Vietnamese) |
| demo_race_a_p___24 | Youth Race (Other Asian) |
| demo_race_a_p___25 | Youth Race (Other Race) |
| demo_ethn_v2 | Youth Ethnicity (Hispanic) |
| demo_prnt_race_a_v2___10 | Caregiver Race (White) |
| demo_prnt_race_a_v2___11 | Caregiver Race (Black) |
| demo_prnt_race_a_v2___12 | Caregiver Race (American Indian) |
| demo_prnt_race_a_v2___13 | Caregiver Race (Alaska Native) |
| demo_prnt_race_a_v2___14 | Caregiver Race (Native Hawaiian) |
| demo_prnt_race_a_v2___15 | Caregiver Race (Guamanian) |
| demo_prnt_race_a_v2___16 | Caregiver Race (Samoan) |
| demo_prnt_race_a_v2___17 | Caregiver Race (Other Pacific Islander) |
| demo_prnt_race_a_v2___18 | Caregiver Race (Asian Indian) |
| demo_prnt_race_a_v2___19 | Caregiver Race (Chinese) |
| demo_prnt_race_a_v2___20 | Caregiver Race (Filipino) |
| demo_prnt_race_a_v2___21 | Caregiver Race (Japanese) |
| demo_prnt_race_a_v2___22 | Caregiver Race (Korean) |
| demo_prnt_race_a_v2___23 | Caregiver Race (Vietnamese) |
| demo_prnt_race_a_v2___24 | Caregiver Race (Other Asian) |
| demo_prnt_race_a_v2___25 | Caregiver Race (Other Race) |
| demo_prnt_ethn_v2 | Caregiver Ethnicity (Hispanic) |
|  |  |
| **Longitudinal Demographics (abcd_lpds01)** |  |
| demo_comb_income_v2_l | Household Income |
| demo_prnt_ed_v2_l | Caregiver 1 Education Level |
| demo_prtnr_ed_v2_l | Caregiver 2 Education Level |
|  |  |
| **Residential History (abcd_rhds01)** |  |
| reshist_addr1_valid | Validity of Primary Residential Address |
| reshist_addr1_adi_wsum | Area Deprivation Index (ADI) Composite Score |
| reshist_addr1_adi_perc | ADI Percentile |
| reshist_addr1_adi_edu_l | ADI: Percentage of population at least 25 years old with less than 9 years of education |
| reshist_addr1_adi_edu_h | ADI: Percentage of population at least 25 years old with at least a high school diploma |
| reshist_addr1_adi_work_c | ADI: Percentage of employed persons at least 16 years old in white-collar occupations |
| reshist_addr1_adi_income | ADI: Median family income |
| reshist_addr1_adi_in_dis | ADI: Income disparity |
| reshist_addr1_adi_home_v | ADI: Median home value |
| reshist_addr1_adi_rent | ADI: Median gross rent |
| reshist_addr1_adi_mortg | ADI: Median monthly mortgage |
| reshist_addr1_adi_home_o | ADI: Percentage of owner-occupied housing units (i.e., home ownership rate) |
| reshist_addr1_adi_crowd | ADI: Percentage of occupied housing units with more than 1 person per room (i.e., crowding) |
| reshist_addr1_adi_unemp | ADI: Percentage of civilian labor force population at least 16 years old who are unemployed (i.e., unemployment rate) |
| reshist_addr1_adi_pov | ADI: Percentage of population below the poverty level |
| reshist_addr1_adi_b138 | ADI: Percentage of population below 138% of the poverty threshold |
| reshist_addr1_adi_sp | ADI: Percentage of single-parent households with children under 18 years old |
| reshist_addr1_adi_ncar | ADI: Percentage of occupied housing units without a motor vehicle |
| reshist_addr1_adi_ntel | ADI: Percentage of occupied housing units without a telephone |
| reshist_addr1_adi_nplumb | ADI: Percentage of occupied housing units without complete plumbing (log) |
|  |  |
| **Longitudinal Tracking (abcd_lt01)** |  |
| site_id_l | Site ID Number |
|  |  |
| **American Community Survey Post Stratification Weights (acspsw03)** | |
| subjectkey | Participant ID |
| rel_family_id | Family ID Number |

**Supplementary Table 2. COVID-19 Questionnaire Variables in Current Analyses**

| **Variable Groupings and Names in Data Release** | **Variable Descriptions [Questionnaire(s) (Q) Including Item]** |
| --- | --- |
| **Demographics and Survey Information** |  |
| interview_age | Youth Age |
| sex | Youth Sex |
| eventname | Questionnaire Number |
|  |  |
| **Disease Burden** |  |
| fam_expose_cv | Was anyone in your household at increased risk for COVID-19 due to work in healthcare or other essential jobs (such as grocery store, factory, gig economy) or use of public transit? [Q1, Q2, Q3] |
| fam_diag_cv | Number of immediate family members (same household) diagnosed with coronavirus [Q2] |
|  |  |
| **Perceived Risk** |  |
| get_coronavirus_cv | I think it is likely that I will get coronavirus [Q1, Q3] |
| think_will_hospitalized_cv | I think it is likely I will be hospitalized or die from the coronavirus [Q1, Q3] |
| think_will_get_cv | I think it is likely that someone very close to me will get coronavirus [Q1, Q3] |
| think_someone_close_get_cv | I think it is likely that someone very close to me will be hospitalized or die from the coronavirus [Q1, Q3] |
|  |  |
| **Youth and Caregiver Worry** |  |
| worry_y_cv | In the past week: How worried have you been about coronavirus (COVID-19)? [Youth Survey] [Q1, Q2, Q3] |
| p_worry_cv | In the LAST week: How worried have you been about coronavirus (COVID-19)? [Caregiver Survey] [Q1, Q2, Q3] |
| child_worry_cv | My child seems worried about becoming ill or that others they know will become ill with coronavirus [Q1, Q3] |
| child_worried_about_cv | My child seems worried about non-health related consequences of coronavirus (e.g., financial) [Q1, Q3] |
|  |  |
| **Caregiver-Youth Communication** |  |
| talk_wash_cv | Please indicate how much you talked about the following with your child in the LAST WEEK - The importance of hand washing for preventing the spread of germs [Q1, Q2, Q3] |
| talk_social_distance_cv | Please indicate how much you talked about the following with your child in the LAST WEEK - The importance of social distancing [Q1, Q2, Q3] |
| talk_cancel_cv | Please indicate how much you talked about the following with your child in the LAST WEEK - Cancellation of school and other events [Q1, Q2, Q3] |
| talk_isolate_cv | Please indicate how much you talked about the following with your child in the LAST WEEK - Avoiding visiting friends or family [Q1, Q2, Q3] |
| talk_symptoms_cv | Please indicate how much you talked about the following with your child in the LAST WEEK - The symptoms of coronavirus [Q1, Q2, Q3] |
| talk_vuln_cv | Please indicate how much you talked about the following with your child in the LAST WEEK - Protecting the elderly or other vulnerable people [Q1, Q2, Q3] |
| talk_mask_cv | Please indicate how much you talked about the following with your child in the LAST WEEK - The importance of wearing a mask [Q3] |
| assure_child_cv | I have told my child that everything will be okay (Note: Referred to as *caregiver reassurance* in main text) [Q1, Q3] |
| encouraged_cv | I have encouraged my child not to focus on coronavirus or its impacts on people and the world (Note: Referred to as *caregiver encouragement* in main text) [Q1, Q3] |
| p_feeling_cv | I discussed with my child my own feelings about coronavirus and its impact on people and the world [Q1, Q3] |
| avoid_talking_about_cv | I have avoided talking to my child about coronavirus [Q1, Q3] |
| p_safety_cv | I have expressed concern to my child that they might not be fully safe from coronavirus [Q1, Q3] |
| prepared_child_cv | I have prepared my child for our lives to change significantly [Q1, Q3] |
|  |  |
| **Youths’ Preventative Actions** | |
| demo_mask_coverage_cv | I wear a mask over my face or protective gear (e.g., gloves, things to cover my clothes) [Q1, Q3] |
| demo_stayed_away_cv | I stay away from people (other than those who live in my house) [Q1, Q3] |
| demo_stayed_indoors_cv | I stay away from people inside my house (e.g., stay in another room or a certain distance away) [Q1, Q3] |
| demo_hand_sanitizer_cv | I use Purell/other hand sanitizer [Q1, Q3] |
| demo_wash_hands_cv | I wash my hands at times other than just after I use the bathroom or before eating [Q1, Q3] |
| demo_wipes_cv | I use Clorox/cleaners to wipe down surfaces [Q1, Q3] |
| demo_touching_things_cv | I avoid touching things (e.g., phone, doorknobs) [Q1, Q3] |
| demo_touching_people_cv | I avoid touching people (e.g., hugging, shaking hands) [Q1, Q3] |

**Supplementary Table 3. Family members diagnosed with COVID-19.**

|  | ***n*** | ***Min*** | ***Mdn*** | **75%** | **95%** | **99%** | ***Max*** | ***Mean*** |
| --- | --- | --- | --- | --- | --- | --- | --- | --- |
| **ADI Decile** |  |  |  |  |  |  |  |  |
| **1** | 735 | 0 | 0 | 0 | 0 | 1 | 2 | 0.04 |
| **2** | 846 | 0 | 0 | 0 | 0 | 2 | 10 | 0.06 |
| **3** | 1,012 | 0 | 0 | 0 | 0 | 2 | 10 | 0.06 |
| **4** | 856 | 0 | 0 | 0 | 0 | 1.9 | 10 | 0.07 |
| **5** | 497 | 0 | 0 | 0 | 0 | 3.5 | 8 | 0.09 |
| **6** | 350 | 0 | 0 | 0 | 0 | 5 | 10 | 0.14 |
| **7** | 290 | 0 | 0 | 0 | 0 | 2.6 | 6 | 0.07 |
| **8** | 218 | 0 | 0 | 0 | 1 | 7.6 | 10 | 0.22 |
| **9** | 202 | 0 | 0 | 0 | 1 | 5 | 10 | 0.18 |
| **10** | 217 | 0 | 0 | 0 | 4 | 7 | 10 | 0.35 |
|  |  |  |  |  |  |  |  |  |
| **Annual Household Income** | | | | | | | | |
| **<$5K** | 96 | 0 | 0 | 0 | 6.7 | 10 | 10 | 0.65 |
| **$5K-$12K** | 120 | 0 | 0 | 0 | 2 | 6.3 | 7 | 0.25 |
| **$12K-$16K** | 96 | 0 | 0 | 0 | 2.7 | 9.1 | 10 | 0.39 |
| **$16K-$25K** | 166 | 0 | 0 | 0 | 1 | 6.5 | 10 | 0.22 |
| **$25K-$35K** | 228 | 0 | 0 | 0 | 1 | 5.7 | 10 | 0.19 |
| **$35K-$50K** | 364 | 0 | 0 | 0 | 1 | 4 | 9 | 0.13 |
| **$50K-$75K** | 707 | 0 | 0 | 0 | 0 | 3.4 | 6 | 0.08 |
| **$75K-$100K** | 773 | 0 | 0 | 0 | 0 | 2.8 | 10 | 0.07 |
| **$100K-$200K** | 1,879 | 0 | 0 | 0 | 0 | 1 | 10 | 0.04 |
| **≥$200K** | 794 | 0 | 0 | 0 | 0 | 1 | 2 | 0.03 |

**Note**: Data are partitioned with respect to ADI decile and percentile of the data for that ADI decile (e.g., ADI Decile = 1, 5% refers to the 5^th^ percentile of the reported number of family members diagnosed with COVID-19 at that ADI decile level). Participants had the option to response 1-9 and 10+, which was coded as “10” for analysis. Mdn = median (50^th^ percentile). K = $1,000 (for income ranges, the upper limit was $1 minus the displayed amount, but was rounded for display purposes).

**Supplementary Table 4. Generalized linear mixed-effects model output for the analysis of caregivers’ responses to, “Was anyone in your household at increased risk for COVID-19 due to work in healthcare or other essential jobs (such as grocery store, factory, gig economy) or use of public transit?”**

|  | *t*(15313) | *p* | *b* | 95% CI |
| --- | --- | --- | --- | --- |
| Intercept | -2.12 | .034 | -0.90 | [-1.73, -0.07] |
| Caregiver Race (American Indian / Alaska Native) | 1.20 | .231 | 1.14 | [-0.73, 3.01] |
| Caregiver Race (Asian) | 0.21 | .830 | 0.11 | [-0.89, 1.11] |
| Caregiver Race (Black) | 0.88 | .380 | 0.39 | [-0.48, 1.26] |
| Caregiver Race (Native Hawaiian / Pacific Islander) | -1.02 | .306 | -1.80 | [-5.24, 1.64] |
| Caregiver Race (Other) | 0.82 | .410 | 0.37 | [-0.51, 1.26] |
| Caregiver Ethnicity | 0.67 | .503 | 0.08 | [-0.15, 0.30] |
| Maximum Caregiver Education | -4.26 | < .001 | -0.48 | [-0.70, -0.26] |
| Questionnaire Number | -0.45 | .652 | -0.02 | [-0.09, 0.06] |
| Family Income | 9.14 | < .001 | 0.46 | [0.36, 0.56] |
| Area Deprivation Index | 10.45 | < .001 | 0.04 | [0.04, 0.05] |
| (Maximum Caregiver Education)^2^ | -6.12 | < .001 | -0.37 | [-0.49, -0.25] |
| (Questionnaire Number)^2^ | 0.79 | .432 | 0.05 | [-0.08, 0.18] |
| (Family Income)^2^ | -6.07 | < .001 | -0.08 | [-0.11, -0.06] |
| (Area Deprivation Index)^2^ | -5.48 | < .001 | -0.001 | [-0.001, -0.0004] |

**Note**: The generalized linear mixed-effects model (distribution = binomial, link = logit) incorporates testing the statistical significance of coefficients against a *t*-distribution. Response options to this item were “No,” “Yes,” “Don’t Know,” and this item was included in questionnaires (Q) 1-3. “Don’t Know” responses were excluded from analysis. “No” was coded as 0; “Yes”, 1. Caregiver Ethnicity was a categorical factor, effect coded with Hispanic/Non-Hispanic as ‑1/+1. Caregiver Race was also a categorical factor, in which “White” served as the reference level. Maximum Caregiver Education (i.e., highest education level between caregivers), Questionnaire Number, Area Deprivation Index, and Family Income were centered continuous factors. The random-effects structure included a random intercept for study site and participant ID. Random effects were restricted to be uncorrelated. Analysis included 15,328 observations across 6,705 participants. The model accounted for 93.6% of the variance in the data (pseudo-*R*^2^ = .936, adjusted pseudo-*R*^2^ = .936).

**Supplementary Table 5. Generalized linear mixed-effects model output for the analysis of caregivers’ responses to, “Number of immediate family members (same household) diagnosed with coronavirus”.**

|  | *t*(5210) | *p* | *b* | 95% CI |
| --- | --- | --- | --- | --- |
| Intercept | -0.24 | .809 | -5.13 | [-46.73, 36.47] |
| Caregiver Race (American Indian / Alaska Native) | -0.12 | .907 | -6.30 | [-112.39, 99.78] |
| Caregiver Race (Asian) | 0.17 | .868 | 3.53 | [-38.08, 45.13] |
| Caregiver Race (Black) | 0.16 | .870 | 3.48 | [-38.12, 45.08] |
| Caregiver Race (Native Hawaiian / Pacific Islander) | -0.06 | .949 | -6.05 | [-189.74, 177.64] |
| Caregiver Race (Other) | 0.12 | .905 | 2.54 | [-39.06, 44.14] |
| Caregiver Ethnicity | -4.22 | < .001 | -0.31 | [-0.45, -0.17] |
| Maximum Caregiver Education | -3.22 | .001 | -0.18 | [-0.30, -0.07] |
| Family Income | -5.52 | < .001 | -0.13 | [-0.18, -0.08] |
| Area Deprivation Index | 3.74 | < .001 | 0.01 | [0.01, 0.02] |
| (Maximum Caregiver Education)^2^ | -3.93 | < .001 | -0.13 | [-0.20, -0.07] |
| (Family Income)^2^ | 2.45 | .014 | 0.02 | [0.004, 0.03] |
| (Area Deprivation Index)^2^ | 1.03 | .303 | 0.0001 | [-0.0001, 0.0002] |

**Note**: The generalized linear mixed-effects model (distribution = Poisson, link = log) incorporates testing the statistical significance of coefficients against a *t*-distribution. Response options to this item were 0 to 10+, and this item was included in questionnaire (Q) 2. The “10+” response option was coded as “10” for analysis. Caregiver Ethnicity was a categorical factor, effect coded with Hispanic/Non-Hispanic as ‑1/+1. Caregiver Race was also a categorical factor, in which “White” served as the reference level. Maximum Caregiver Education (i.e., highest education level between caregivers), Area Deprivation Index, and Family Income were centered continuous factors. The random-effects structure included a random intercept for study site. Analysis included 5,223 observations across 5,223 participants. The model accounted for 3.0% of the variance in the data (pseudo-*R*^2^ = .030, adjusted pseudo-*R*^2^ = .028).

**Supplementary Table 6. Linear mixed-effects model output for the analysis of all caregivers’ responses to, “I think it is likely that I will get coronavirus.”**

|  | *t*(10081) | *p* | *b* | 95% CI |
| --- | --- | --- | --- | --- |
| Intercept | 34.26 | < .001 | 2.52 | [2.38, 2.67] |
| Caregiver Race (American Indian / Alaska Native) | -0.96 | .337 | -0.15 | [-0.46, 0.16] |
| Caregiver Race (Asian) | 0.30 | .764 | 0.02 | [-0.13, 0.18] |
| Caregiver Race (Black) | -3.78 | < .001 | -0.27 | [-0.41, -0.13] |
| Caregiver Race (Native Hawaiian / Pacific Islander) | 0.99 | .322 | 0.28 | [-0.27, 0.82] |
| Caregiver Race (Other) | 0.57 | .570 | 0.04 | [-0.10, 0.18] |
| Caregiver Ethnicity | 0.99 | .324 | 0.02 | [-0.02, 0.06] |
| Maximum Caregiver Education | 5.41 | < .001 | 0.10 | [0.06, 0.13] |
| Questionnaire Number | 14.44 | < .001 | 0.10 | [0.09, 0.12] |
| Family Income | 5.42 | < .001 | 0.04 | [0.03, 0.06] |
| Area Deprivation Index | -2.57 | .010 | -0.002 | [-0.003, -0.0005] |
| (Maximum Caregiver Education)^2^ | -0.92 | .360 | -0.01 | [-0.03, 0.01] |
| (Family Income)^2^ | 1.13 | .260 | 0.002 | [-0.002, 0.01] |
| (Area Deprivation Index)^2^ | 1.03 | .305 | 0.00002 | [-0.00002, 0.0001] |

**Note**: The linear mixed-effects model incorporates testing the statistical significance of coefficients against a *t*-distribution. Response options to this item were on a 5-point Likert scale: 1 = Strongly Disagree, 2 = Disagree, 3 = Neither Disagree or Agree, 4 = Agree, 5 = Strongly Agree. This item was included in questionnaires (Q) 1 and 3. Caregiver Ethnicity was a categorical factor, effect coded with Hispanic/Non-Hispanic as ‑1/+1. Caregiver Race was also a categorical factor, in which “White” served as the reference level. Maximum Caregiver Education (i.e., highest education level between caregivers), Questionnaire Number, Area Deprivation Index, and Family Income were centered continuous factors. The random-effects structure included a random intercept for study site and participant ID. Random effects were restricted to be uncorrelated. Analysis included 10,095 observations across 6,211 participants. The model accounted for 53.3% of the variance in the data (*R*^2^ = .533, adjusted *R*^2^ = .532).

**Supplementary Table 7. Linear mixed-effects model output for the analysis of all caregivers’ responses to, “I think it is likely I will be hospitalized or die from the coronavirus.”**

|  | *t*(10080) | *p* | *b* | 95% CI |
| --- | --- | --- | --- | --- |
| Intercept | 35.56 | < .001 | 2.23 | [2.11, 2.35] |
| Caregiver Race (American Indian / Alaska Native) | 0.04 | .964 | 0.01 | [-0.28, 0.29] |
| Caregiver Race (Asian) | -0.37 | .708 | -0.03 | [-0.17, 0.11] |
| Caregiver Race (Black) | -4.55 | < .001 | -0.30 | [-0.43, -0.17] |
| Caregiver Race (Native Hawaiian / Pacific Islander) | 2.97 | .003 | 0.76 | [0.26, 1.26] |
| Caregiver Race (Other) | -2.64 | .008 | -0.17 | [-0.30, -0.05] |
| Caregiver Ethnicity | -1.62 | .104 | -0.03 | [-0.06, 0.01] |
| Maximum Caregiver Education | 0.83 | .406 | 0.01 | [-0.02, 0.05] |
| Questionnaire Number | 13.08 | < .001 | 0.08 | [0.07, 0.09] |
| Family Income | -1.53 | .126 | -0.01 | [-0.02, 0.00] |
| Area Deprivation Index | -0.41 | .684 | -0.0003 | [-0.001, 0.001] |
| (Maximum Caregiver Education)^2^ | -0.77 | .441 | -0.01 | [-0.02, 0.01] |
| (Family Income)^2^ | -1.53 | .125 | -0.003 | [-0.01, 0.001] |
| (Area Deprivation Index)^2^ | 0.65 | .513 | 0.00001 | [-0.00002, 0.00004] |

**Note**: The linear mixed-effects model incorporates testing the statistical significance of coefficients against a *t*-distribution. Response options to this item were on a 5-point Likert scale: 1 = Strongly Disagree, 2 = Disagree, 3 = Neither Disagree or Agree, 4 = Agree, 5 = Strongly Agree. This item was included in questionnaires (Q) 1 and 3. Caregiver Ethnicity was a categorical factor, effect coded with Hispanic/Non-Hispanic as ‑1/+1. Caregiver Race was also a categorical factor, in which “White” served as the reference level. Maximum Caregiver Education (i.e., highest education level between caregivers), Questionnaire Number, Area Deprivation Index, and Family Income were centered continuous factors. The random-effects structure included a random intercept for study site and participant ID. Random effects were restricted to be uncorrelated. Analysis included 10,094 observations across 6,211 participants. The model accounted for 55.7% of the variance in the data (*R*^2^ = .557, adjusted *R*^2^ = .557).

**Supplementary Table 8. Linear mixed-effects model output for the analysis of all caregivers’ responses to, “I think it is likely that someone very close to me will get coronavirus.”**

|  | *t*(10082) | *p* | *b* | 95% CI |
| --- | --- | --- | --- | --- |
| Intercept | 35.05 | < .001 | 2.84 | [2.69, 3.00] |
| Caregiver Race (American Indian / Alaska Native) | -0.47 | .637 | -0.08 | [-0.43, 0.26] |
| Caregiver Race (Asian) | -3.39 | .001 | -0.30 | [-0.47, -0.12] |
| Caregiver Race (Black) | -4.02 | < .001 | -0.32 | [-0.48, -0.16] |
| Caregiver Race (Native Hawaiian / Pacific Islander) | 1.87 | .061 | 0.58 | [-0.03, 1.19] |
| Caregiver Race (Other) | 1.08 | .282 | 0.09 | [-0.07, 0.24] |
| Caregiver Ethnicity | 3.00 | .003 | 0.07 | [0.02, 0.11] |
| Maximum Caregiver Education | 7.73 | < .001 | 0.15 | [0.12, 0.19] |
| Questionnaire Number | 17.73 | < .001 | 0.14 | [0.12, 0.15] |
| Family Income | 7.10 | < .001 | 0.06 | [0.04, 0.08] |
| Area Deprivation Index | -2.07 | .038 | -0.002 | [-0.003, -0.0001] |
| (Maximum Caregiver Education)^2^ | -1.53 | .125 | -0.02 | [-0.04, 0.004] |
| (Family Income)^2^ | 1.92 | .055 | 0.004 | [-0.0001, 0.01] |
| (Area Deprivation Index)^2^ | 1.75 | .081 | 0.00003 | [-0.000004, 0.0001] |

**Note**: The linear mixed-effects model incorporates testing the statistical significance of coefficients against a *t*-distribution. Response options to this item were on a 5-point Likert scale: 1 = Strongly Disagree, 2 = Disagree, 3 = Neither Disagree or Agree, 4 = Agree, 5 = Strongly Agree. This item was included in questionnaires (Q) 1 and 3. Caregiver Ethnicity was a categorical factor, effect coded with Hispanic/Non-Hispanic as ‑1/+1. Caregiver Race was also a categorical factor, in which “White” served as the reference level. Maximum Caregiver Education (i.e., highest education level between caregivers), Questionnaire Number, Area Deprivation Index, and Family Income were centered continuous factors. The random-effects structure included a random intercept for study site and participant ID. Random effects were restricted to be uncorrelated. Analysis included 10,096 observations across 6,211 participants. The model accounted for 58.7% of the variance in the data (*R*^2^ = .587, adjusted *R*^2^ = .586).

**Supplementary Table 9. Linear mixed-effects model output for the analysis of all caregivers’ responses to, “I think it is likely that someone very close to me will be hospitalized or die from the coronavirus.”**

|  | *t*(10082) | *p* | *b* | 95% CI |
| --- | --- | --- | --- | --- |
| Intercept | 34.19 | < .001 | 2.58 | [2.43, 2.72] |
| Caregiver Race (American Indian / Alaska Native) | -0.43 | .668 | -0.07 | [-0.41, 0.26] |
| Caregiver Race (Asian) | -2.82 | .005 | -0.24 | [-0.41, -0.07] |
| Caregiver Race (Black) | -3.29 | .001 | -0.26 | [-0.41, -0.10] |
| Caregiver Race (Native Hawaiian / Pacific Islander) | 2.49 | .013 | 0.75 | [0.16, 1.35] |
| Caregiver Race (Other) | -0.36 | .719 | -0.03 | [-0.18, 0.13] |
| Caregiver Ethnicity | 1.15 | .250 | 0.02 | [-0.02, 0.06] |
| Maximum Caregiver Education | 4.32 | < .001 | 0.08 | [0.05, 0.12] |
| Questionnaire Number | 10.30 | < .001 | 0.08 | [0.06, 0.09] |
| Family Income | 2.96 | .003 | 0.02 | [0.01, 0.04] |
| Area Deprivation Index | -0.69 | .491 | -0.001 | [-0.002, 0.001] |
| (Maximum Caregiver Education)^2^ | -0.27 | .783 | -0.003 | [-0.02, 0.02] |
| (Family Income)^2^ | 0.13 | .893 | 0.0003 | [-0.004, 0.01] |
| (Area Deprivation Index)^2^ | 0.81 | .417 | 0.00002 | [-0.00002, 0.0001] |

**Note**: The linear mixed-effects model incorporates testing the statistical significance of coefficients against a *t*-distribution. Response options to this item were on a 5-point Likert scale: 1 = Strongly Disagree, 2 = Disagree, 3 = Neither Disagree or Agree, 4 = Agree, 5 = Strongly Agree. This item was included in questionnaires (Q) 1 and 3. Caregiver Ethnicity was a categorical factor, effect coded with Hispanic/Non-Hispanic as ‑1/+1. Caregiver Race was also a categorical factor, in which “White” served as the reference level. Maximum Caregiver Education (i.e., highest education level between caregivers), Questionnaire Number, Area Deprivation Index, and Family Income were centered continuous factors. The random-effects structure included a random intercept for study site and participant ID. Random effects were restricted to be uncorrelated. Analysis included 10,096 observations across 6,211 participants. The model accounted for 52.7% of the variance in the data (*R*^2^ = .527, adjusted *R*^2^ = .526).

**Supplementary Table 10. Linear mixed-effects model output for the analysis of caregivers’ responses to, “I think it is likely that I will get coronavirus,” for caregivers who did not report any household member had been diagnosed with COVID-19.**

|  | *t*(7992) | *p* | *b* | 95% CI |
| --- | --- | --- | --- | --- |
| Intercept | 29.34 | < .001 | 2.46 | [2.30, 2.63] |
| Caregiver Race (American Indian / Alaska Native) | -0.48 | .629 | -0.09 | [-0.46, 0.28] |
| Caregiver Race (Asian) | 0.61 | .539 | 0.05 | [-0.12, 0.23] |
| Caregiver Race (Black) | -4.04 | < .001 | -0.34 | [-0.51, -0.18] |
| Caregiver Race (Native Hawaiian / Pacific Islander) | 0.39 | .695 | 0.13 | [-0.51, 0.77] |
| Caregiver Race (Other) | 1.42 | .155 | 0.12 | [-0.05, 0.29] |
| Caregiver Ethnicity | 2.43 | .015 | 0.06 | [0.01, 0.10] |
| Maximum Caregiver Education | 5.54 | < .001 | 0.12 | [0.08, 0.16] |
| Questionnaire Number | 12.62 | < .001 | 0.10 | [0.08, 0.11] |
| Family Income | 4.22 | < .001 | 0.04 | [0.02, 0.06] |
| Area Deprivation Index | -1.64 | .102 | -0.001 | [-0.003, 0.0003] |
| (Maximum Caregiver Education)^2^ | -2.09 | .036 | -0.02 | [-0.05, -0.001] |
| (Family Income)^2^ | 0.84 | .399 | 0.002 | [-0.003, 0.01] |
| (Area Deprivation Index)^2^ | 0.40 | .692 | 0.00001 | [-0.00003, 0.00005] |

**Note**: The linear mixed-effects model incorporates testing the statistical significance of coefficients against a *t*-distribution. Response options to this item were on a 5-point Likert scale: 1 = Strongly Disagree, 2 = Disagree, 3 = Neither Disagree or Agree, 4 = Agree, 5 = Strongly Agree. This item was included in questionnaires (Q) 1 and 3. Caregiver Ethnicity was a categorical factor, effect coded with Hispanic/Non-Hispanic as ‑1/+1. Caregiver Race was also a categorical factor, in which “White” served as the reference level. Maximum Caregiver Education (i.e., highest education level between caregivers), Questionnaire Number, Area Deprivation Index, and Family Income were centered continuous factors. The random-effects structure included a random intercept for study site and participant ID. Random effects were restricted to be uncorrelated. Analysis included 8.006 observations across 4,597 participants. The model accounted for 53.4% of the variance in the data (*R*^2^ = .534, adjusted *R*^2^ = .533).

**Supplementary Table 11. Linear mixed-effects model output for the analysis of all caregivers’ responses to, “I think it is likely I will be hospitalized or die from the coronavirus,” for caregivers who did not report any household member had been diagnosed with COVID-19.**

|  | *t*(7991) | *p* | *b* | 95% CI |
| --- | --- | --- | --- | --- |
| Intercept | 29.82 | < .001 | 2.17 | [2.03, 2.32] |
| Caregiver Race (American Indian / Alaska Native) | 0.55 | .584 | 0.09 | [-0.24, 0.43] |
| Caregiver Race (Asian) | -0.42 | .674 | -0.03 | [-0.19, 0.12] |
| Caregiver Race (Black) | -4.37 | < .001 | -0.34 | [-0.49, -0.19] |
| Caregiver Race (Native Hawaiian / Pacific Islander) | 2.12 | .034 | 0.63 | [0.05, 1.21] |
| Caregiver Race (Other) | -1.61 | .107 | -0.12 | [-0.28, 0.03] |
| Caregiver Ethnicity | -0.67 | .500 | -0.01 | [-0.05, 0.03] |
| Maximum Caregiver Education | 1.86 | .062 | 0.04 | [-0.002, 0.08] |
| Questionnaire Number | 12.76 | < .001 | 0.08 | [0.07, 0.10] |
| Family Income | -0.76 | .445 | -0.01 | [-0.02, 0.01] |
| Area Deprivation Index | -0.35 | .725 | -0.0002 | [-0.002, 0.001] |
| (Maximum Caregiver Education)^2^ | -2.37 | .018 | -0.02 | [-0.04, -0.004] |
| (Family Income)^2^ | -1.10 | .271 | -0.002 | [-0.01, 0.002] |
| (Area Deprivation Index)^2^ | 1.02 | .309 | 0.00002 | [-0.00002, 0.0001] |

**Note**: The linear mixed-effects model incorporates testing the statistical significance of coefficients against a *t*-distribution. Response options to this item were on a 5-point Likert scale: 1 = Strongly Disagree, 2 = Disagree, 3 = Neither Disagree or Agree, 4 = Agree, 5 = Strongly Agree. This item was included in questionnaires (Q) 1 and 3. Caregiver Ethnicity was a categorical factor, effect coded with Hispanic/Non-Hispanic as ‑1/+1. Caregiver Race was also a categorical factor, in which “White” served as the reference level. Maximum Caregiver Education (i.e., highest education level between caregivers), Questionnaire Number, Area Deprivation Index, and Family Income were centered continuous factors. The random-effects structure included a random intercept for study site and participant ID. Random effects were restricted to be uncorrelated. Analysis included 8,005 observations across 4,597 participants. The model accounted for 57.1% of the variance in the data (*R*^2^ = .571, adjusted *R*^2^ = .571).

**Supplementary Table 12. Linear mixed-effects model output for the analysis of all caregivers’ responses to, “I think it is likely that someone very close to me will get coronavirus,” for caregivers who did not report any household member had been diagnosed with COVID-19.**

|  | *t*(7993) | *p* | *b* | 95% CI |
| --- | --- | --- | --- | --- |
| Intercept | 29.23 | < .001 | 2.74 | [2.56, 2.92] |
| Caregiver Race (American Indian / Alaska Native) | 0.05 | .964 | 0.01 | [-0.41, 0.43] |
| Caregiver Race (Asian) | -2.36 | .018 | -0.24 | [-0.43, -0.04] |
| Caregiver Race (Black) | -3.26 | .001 | -0.31 | [-0.50, -0.12] |
| Caregiver Race (Native Hawaiian / Pacific Islander) | 0.67 | .505 | 0.24 | [-0.47, 0.96] |
| Caregiver Race (Other) | 1.90 | .058 | 0.18 | [-0.01, 0.37] |
| Caregiver Ethnicity | 4.04 | < .001 | 0.10 | [0.05, 0.15] |
| Maximum Caregiver Education | 7.27 | < .001 | 0.18 | [0.13, 0.22] |
| Questionnaire Number | 15.25 | < .001 | 0.13 | [0.11, 0.15] |
| Family Income | 5.59 | < .001 | 0.06 | [0.04, 0.08] |
| Area Deprivation Index | -1.69 | .092 | -0.002 | [-0.003, 0.0003] |
| (Maximum Caregiver Education)^2^ | -2.43 | .015 | -0.03 | [-0.05, -0.01] |
| (Family Income)^2^ | 1.63 | .104 | 0.004 | [-0.001, 0.01] |
| (Area Deprivation Index)^2^ | 1.64 | .102 | 0.00004 | [-0.00001, 0.0001] |

**Note**: The linear mixed-effects model incorporates testing the statistical significance of coefficients against a *t*-distribution. Response options to this item were on a 5-point Likert scale: 1 = Strongly Disagree, 2 = Disagree, 3 = Neither Disagree or Agree, 4 = Agree, 5 = Strongly Agree. This item was included in questionnaires (Q) 1 and 3. Caregiver Ethnicity was a categorical factor, effect coded with Hispanic/Non-Hispanic as ‑1/+1. Caregiver Race was also a categorical factor, in which “White” served as the reference level. Maximum Caregiver Education (i.e., highest education level between caregivers), Questionnaire Number, Area Deprivation Index, and Family Income were centered continuous factors. The random-effects structure included a random intercept for study site and participant ID. Random effects were restricted to be uncorrelated. Analysis included 8,007 observations across 4,597 participants. The model accounted for 58.7% of the variance in the data (*R*^2^ = .587, adjusted *R*^2^ = .587).

**Supplementary Table 13. Linear mixed-effects model output for the analysis of all caregivers’ responses to, “I think it is likely that someone very close to me will be hospitalized or die from the coronavirus,” for caregivers who did not report any household member had been diagnosed with COVID-19.**

|  | *t*(7993) | *p* | *b* | 95% CI |
| --- | --- | --- | --- | --- |
| Intercept | 28.28 | < .001 | 2.49 | [2.31, 2.66] |
| Caregiver Race (American Indian / Alaska Native) | -0.04 | .968 | -0.01 | [-0.41, 0.40] |
| Caregiver Race (Asian) | -1.99 | .047 | -0.19 | [-0.38, -0.003] |
| Caregiver Race (Black) | -2.67 | .007 | -0.25 | [-0.43, -0.07] |
| Caregiver Race (Native Hawaiian / Pacific Islander) | 1.37 | .172 | 0.49 | [-0.21, 1.18] |
| Caregiver Race (Other) | 0.55 | .583 | 0.05 | [-0.13, 0.23] |
| Caregiver Ethnicity | 1.61 | .108 | 0.04 | [-0.01, 0.09] |
| Maximum Caregiver Education | 4.83 | < .001 | 0.11 | [0.07, 0.16] |
| Questionnaire Number | 9.26 | < .001 | 0.08 | [0.06, 0.09] |
| Family Income | 2.10 | .036 | 0.02 | [0.001, 0.04] |
| Area Deprivation Index | -0.53 | .598 | -0.0004 | [-0.002, 0.001] |
| (Maximum Caregiver Education)^2^ | -1.58 | .114 | -0.02 | [-0.04, 0.01] |
| (Family Income)^2^ | 0.49 | .624 | 0.001 | [-0.004, 0.01] |
| (Area Deprivation Index)^2^ | 1.23 | .219 | 0.00003 | [-0.00002, 0.0001] |

**Note**: The linear mixed-effects model incorporates testing the statistical significance of coefficients against a *t*-distribution. Response options to this item were on a 5-point Likert scale: 1 = Strongly Disagree, 2 = Disagree, 3 = Neither Disagree or Agree, 4 = Agree, 5 = Strongly Agree. This item was included in questionnaires (Q) 1 and 3. Caregiver Ethnicity was a categorical factor, effect coded with Hispanic/Non-Hispanic as ‑1/+1. Caregiver Race was also a categorical factor, in which “White” served as the reference level. Maximum Caregiver Education (i.e., highest education level between caregivers), Questionnaire Number, Area Deprivation Index, and Family Income were centered continuous factors. The random-effects structure included a random intercept for study site and participant ID. Random effects were restricted to be uncorrelated. Analysis included 8,007 observations across 4,597 participants. The model accounted for 54.7% of the variance in the data (*R*^2^ = .547, adjusted *R*^2^ = .546).

**Supplementary Table 14. Linear mixed-effects model output for the analysis of caregivers’ responses to, “In the LAST week: How worried have you been about coronavirus (COVID-19)?”**

|  | *t*(15357) | *p* | *b* | 95% CI |
| --- | --- | --- | --- | --- |
| Intercept | 47.77 | < .001 | 3.37 | [3.23, 3.51] |
| Caregiver Race (American Indian / Alaska Native) | -1.69 | .091 | -0.26 | [-0.56, 0.04] |
| Caregiver Race (Asian) | -3.32 | .001 | -0.26 | [-0.41, -0.11] |
| Caregiver Race (Black) | 1.48 | .140 | 0.10 | [-0.03, 0.24] |
| Caregiver Race (Native Hawaiian / Pacific Islander) | 3.64 | < .001 | 0.97 | [0.45, 1.50] |
| Caregiver Race (Other) | -2.76 | .006 | -0.19 | [-0.33, -0.06] |
| Caregiver Ethnicity | -5.60 | < .001 | -0.11 | [-0.15, -0.07] |
| Maximum Caregiver Education | -0.63 | .529 | -0.01 | [-0.05, 0.02] |
| Questionnaire Number | -15.17 | < .001 | -0.09 | [-0.11, -0.08] |
| Family Income | -2.87 | .004 | -0.02 | [-0.04, -0.01] |
| Area Deprivation Index | -3.08 | .002 | -0.002 | [-0.004, -0.001] |
| (Maximum Caregiver Education)^2^ | 2.41 | .016 | 0.02 | [0.004, 0.04] |
| (Questionnaire Number)^2^ | -3.86 | < .001 | -0.04 | [-0.06, -0.02] |
| (Family Income)^2^ | 1.32 | .187 | 0.003 | [-0.001, 0.01] |
| (Area Deprivation Index)^2^ | 2.05 | .041 | 0.00004 | [0.000002, 0.0001] |

**Note**: The linear mixed-effects model incorporates testing the statistical significance of coefficients against a *t*-distribution. Response options to this item were on a 5-point Likert scale: 1 = Not at all, 2 = Slightly, 3 = Moderately, 4 = Very, 5 = Extremely. This item was included in all questionnaires (Q1-3). Caregiver Ethnicity was a categorical factor, effect coded with Hispanic/Non-Hispanic as ‑1/+1. Caregiver Race was also a categorical factor, in which “White” served as the reference level. Maximum Caregiver Education (i.e., highest education level between caregivers), Questionnaire Number, Area Deprivation Index, and Family Income were centered continuous factors. The random-effects structure included a random intercept for study site and participant ID. Random effects were restricted to be uncorrelated. Analysis included 15,372 observations across 6,713 participants. The model accounted for 71.4% of the variance in the data (*R*^2^ = .714, adjusted *R*^2^ = .714).

**Supplementary Table 15. Linear mixed-effects model output for the analysis of youths’ responses to, “In the past week: How worried have you been about coronavirus (COVID-19)?”**

|  | *t*(12510) | *p* | *b* | 95% CI |
| --- | --- | --- | --- | --- |
| Intercept | 32.82 | < .001 | 2.44 | [2.29, 2.58] |
| Youth Race (American Indian / Alaska Native) | -0.95 | .340 | -0.17 | [-0.51, 0.18] |
| Youth Race (Asian) | 0.55 | .581 | 0.05 | [-0.12, 0.22] |
| Youth Race (Black) | 2.57 | .010 | 0.19 | [0.05, 0.34] |
| Youth Race (Native Hawaiian / Pacific Islander) | 0.49 | .624 | 0.14 | [-0.42, 0.70] |
| Youth Race (Other) | -0.58 | .563 | -0.04 | [-0.18, 0.10] |
| Youth Ethnicity | -3.11 | .002 | -0.06 | [-0.09, -0.02] |
| Maximum Caregiver Education | 1.08 | .279 | 0.02 | [-0.02, 0.06] |
| Questionnaire Number | -7.56 | < .001 | -0.06 | [-0.07, -0.04] |
| Family Income | -2.32 | .020 | -0.02 | [-0.03, -0.003] |
| Area Deprivation Index | -1.80 | .072 | -0.001 | [-0.003, 0.0001] |
| Youth Age | 0.21 | .831 | 0.0002 | [-0.002, 0.003] |
| Youth Sex | 8.65 | < .001 | 0.10 | [0.08, 0.12] |
| (Maximum Caregiver Education)^2^ | 0.67 | .502 | 0.01 | [-0.01, 0.03] |
| (Questionnaire Number)^2^ | 0.21 | .834 | 0.003 | [-0.02, 0.03] |
| (Family Income)^2^ | 2.90 | .004 | 0.01 | [0.002, 0.01] |
| (Area Deprivation Index)^2^ | 1.17 | .242 | 0.00002 | [-0.00001, 0.0001] |

**Note**: The linear mixed-effects model incorporates testing the statistical significance of coefficients against a *t*-distribution. Response options to this item were on a 5-point Likert scale: 1 = Not at all, 2 = Slightly, 3 = Moderately, 4 = Very, 5 = Extremely. This item was included in all questionnaires (Q1-3). Youth Ethnicity was a categorical factor, effect coded with Hispanic/Non-Hispanic as ‑1/+1. Youth Sex was also a categorical factor, effects coded with Male/Female as -1/+1. Youth Race was also a categorical factor, in which “White” served as the reference level. Maximum Caregiver Education (i.e., highest education level between caregivers), Questionnaire Number, Area Deprivation Index, Youth Age, and Family Income were centered continuous factors. The random-effects structure included a random intercept for study site and participant ID. Random effects were restricted to be uncorrelated. Analysis included 12,527 observations across 5,890 participants. The model accounted for 61.0% of the variance in the data (*R*^2^ = .610, adjusted *R*^2^ = .610).

**Supplementary Table 16. Linear mixed-effects model output for the analysis of caregivers’ responses to, “My child seems worried about becoming ill or that others they know will become ill with coronavirus.”**

|  | *t*(9842) | *p* | *b* | 95% CI |
| --- | --- | --- | --- | --- |
| Intercept | 32.36 | < .001 | 2.51 | [2.36, 2.66] |
| Youth Race (American Indian / Alaska Native) | 0.18 | .858 | 0.03 | [-0.33, 0.40] |
| Youth Race (Asian) | -0.04 | .971 | -0.003 | [-0.19, 0.18] |
| Youth Race (Black) | -0.18 | .853 | -0.01 | [-0.17, 0.14] |
| Youth Race (Native Hawaiian / Pacific Islander) | 0.87 | .387 | 0.27 | [-0.34, 0.88] |
| Youth Race (Other) | -0.72 | .471 | -0.06 | [-0.21, 0.10] |
| Youth Ethnicity | -5.24 | < .001 | -0.10 | [-0.13, -0.06] |
| Maximum Caregiver Education | -2.38 | .017 | -0.05 | [-0.09, -0.01] |
| Questionnaire Number | 7.88 | < .001 | 0.06 | [0.05, 0.08] |
| Family Income | -4.62 | < .001 | -0.04 | [-0.06, -0.02] |
| Area Deprivation Index | -0.33 | .741 | -0.0003 | [-0.002, 0.001] |
| Youth Age | -2.42 | .015 | -0.003 | [-0.01, -0.001] |
| Youth Sex | 0.55 | .579 | 0.01 | [-0.02, 0.03] |
| (Maximum Caregiver Education)^2^ | 2.94 | .003 | 0.03 | [0.01, 0.05] |
| (Family Income)^2^ | -0.0001 | 1.000 | -0.0000003 | [-0.01, 0.01] |
| (Area Deprivation Index)^2^ | 0.73 | .468 | 0.00001 | [-0.00002, 0.0001] |

**Note**: The linear mixed-effects model incorporates testing the statistical significance of coefficients against a *t*-distribution. Response options to this item were on a 5-point Likert scale: 1 = Strongly Disagree, 2 = Disagree, 3 = Neither Disagree or Agree, 4 = Agree, 5 = Strongly Agree. This item was included in questionnaires (Q) 1 and 3. Youth Ethnicity was a categorical factor, effect coded with Hispanic/Non-Hispanic as ‑1/+1. Youth Sex was also a categorical factor, effects coded with Male/Female as -1/+1. Youth Race was also a categorical factor, in which “White” served as the reference level. Maximum Caregiver Education (i.e., highest education level between caregivers), Questionnaire Number, Area Deprivation Index, Youth Age, and Family Income were centered continuous factors. The random-effects structure included a random intercept for study site and participant ID. Random effects were restricted to be uncorrelated. Analysis included 9,858 observations across 6,072 participants. The model accounted for 52.6% of the variance in the data (*R*^2^ = .526, adjusted *R*^2^ = .526).

**Supplementary Table 17. Linear mixed-effects model output for the analysis of caregivers’ responses to, “My child seems worried about non-health related consequences of coronavirus (e.g., financial).”**

|  | *t*(9840) | *p* | *b* | 95% CI |
| --- | --- | --- | --- | --- |
| Intercept | 32.71 | < .001 | 2.46 | [2.31, 2.60] |
| Youth Race (American Indian / Alaska Native) | 1.14 | .255 | 0.21 | [-0.15, 0.56] |
| Youth Race (Asian) | -0.71 | .480 | -0.06 | [-0.24, 0.11] |
| Youth Race (Black) | -1.98 | .048 | -0.15 | [-0.31, 0.00] |
| Youth Race (Native Hawaiian / Pacific Islander) | 0.48 | .632 | 0.14 | [-0.44, 0.73] |
| Youth Race (Other) | -0.20 | .840 | -0.01 | [-0.16, 0.13] |
| Youth Ethnicity | -1.23 | .220 | -0.02 | [-0.06, 0.01] |
| Maximum Caregiver Education | -1.43 | .152 | -0.03 | [-0.07, 0.01] |
| Questionnaire Number | 2.08 | .037 | 0.02 | [0.001, 0.03] |
| Family Income | -8.54 | < .001 | -0.07 | [-0.09, -0.05] |
| Area Deprivation Index | -0.95 | .342 | -0.001 | [-0.002, 0.001] |
| Youth Age | 1.04 | .300 | 0.001 | [-0.001, 0.004] |
| Youth Sex | 1.89 | .059 | 0.02 | [-0.001, 0.05] |
| (Maximum Caregiver Education)^2^ | 1.24 | .217 | 0.01 | [-0.01, 0.03] |
| (Family Income)^2^ | -1.24 | .214 | -0.003 | [-0.01, 0.002] |
| (Area Deprivation Index)^2^ | 0.66 | .506 | 0.00001 | [-0.00002, 0.00005] |

**Note**: The linear mixed-effects model incorporates testing the statistical significance of coefficients against a *t*-distribution. Response options to this item were on a 5-point Likert scale: 1 = Strongly Disagree, 2 = Disagree, 3 = Neither Disagree or Agree, 4 = Agree, 5 = Strongly Agree. This item was included in questionnaires (Q) 1 and 3. Youth Ethnicity was a categorical factor, effect coded with Hispanic/Non-Hispanic as ‑1/+1. Youth Sex was also a categorical factor, effects coded with Male/Female as -1/+1. Youth Race was also a categorical factor, in which “White” served as the reference level. Maximum Caregiver Education (i.e., highest education level between caregivers), Questionnaire Number, Area Deprivation Index, Youth Age, and Family Income were centered continuous factors. The random-effects structure included a random intercept for study site and participant ID. Random effects were restricted to be uncorrelated. Analysis included 9,856 observations across 6,070 participants. The model accounted for 45.7% of the variance in the data (*R*^2^ = .457, adjusted *R*^2^ = .456).

**Supplementary Table 18. Linear mixed-effects model output for the analysis of caregivers’ responses to, “In the LAST week: How worried have you been about coronavirus (COVID-19)?” including the factor referring to increased household risk.**

|  | *t*(15312) | *p* | *b* | 95% CI |
| --- | --- | --- | --- | --- |
| Intercept | 47.83 | < .001 | 3.37 | [3.24, 3.51] |
| Caregiver Race (American Indian / Alaska Native) | -1.76 | .078 | -0.27 | [-0.57, 0.03] |
| Caregiver Race (Asian) | -3.32 | .001 | -0.26 | [-0.41, -0.10] |
| Caregiver Race (Black) | 1.41 | .158 | 0.10 | [-0.04, 0.23] |
| Caregiver Race (Native Hawaiian / Pacific Islander) | 3.68 | < .001 | 0.98 | [0.46, 1.51] |
| Caregiver Race (Other) | -2.71 | .007 | -0.19 | [-0.33, -0.05] |
| Caregiver Ethnicity | -5.64 | < .001 | -0.11 | [-0.15, -0.07] |
| Maximum Caregiver Education | -0.57 | .569 | -0.01 | [-0.04, 0.02] |
| Questionnaire Number | -15.05 | < .001 | -0.09 | [-0.11, -0.08] |
| Family Income | -3.19 | .001 | -0.02 | [-0.04, -0.01] |
| Area Deprivation Index | -3.29 | .001 | -0.002 | [-0.004, -0.001] |
| Increased Risk due to Job/Transit | -3.34 | .001 | -0.03 | [-0.05, -0.01] |
| (Maximum Caregiver Education)^2^ | 2.58 | .010 | 0.02 | [0.01, 0.04] |
| (Questionnaire Number)^2^ | -3.84 | < .001 | -0.04 | [-0.06, -0.02] |
| (Family Income)^2^ | 1.58 | .114 | 0.003 | [-0.001, 0.01] |
| (Area Deprivation Index)^2^ | 2.19 | .029 | 0.00004 | [0.000004, 0.0001] |

**Note**: The linear mixed-effects model incorporates testing the statistical significance of coefficients against a *t*-distribution. Response options to this item were on a 5-point Likert scale: 1 = Not at all, 2 = Slightly, 3 = Moderately, 4 = Very, 5 = Extremely. This item was included in all questionnaires (Q1-3). Caregiver Ethnicity was a categorical factor, effect coded with Hispanic/Non-Hispanic as ‑1/+1. Increase Risk due to Job/Transit was also a categorical factor, effects coded with Risk/No-Risk as -1/+1. Caregiver Race was also a categorical factor, in which “White” served as the reference level. Maximum Caregiver Education (i.e., highest education level between caregivers), Questionnaire Number, Area Deprivation Index, and Family Income were centered continuous factors. The random-effects structure included a random intercept for study site and participant ID. Random effects were restricted to be uncorrelated. Analysis included 15,328 observations across 6,705 participants. The model accounted for 71.3% of the variance in the data (*R*^2^ = .713, adjusted *R*^2^ = .713).

**Supplementary Table 19. Linear mixed-effects model output for the analysis of youths’ responses to, “In the past week: How worried have you been about coronavirus (COVID-19)?” including the factor referring to increased household risk.**

|  | *t*(11880) | *p* | *b* | 95% CI |
| --- | --- | --- | --- | --- |
| Intercept | 29.66 | < .001 | 2.44 | [2.28, 2.60] |
| Youth Race (American Indian / Alaska Native) | -0.73 | .463 | -0.14 | [-0.51, 0.23] |
| Youth Race (Asian) | 0.54 | .586 | 0.05 | [-0.13, 0.24] |
| Youth Race (Black) | 2.42 | .016 | 0.20 | [0.04, 0.36] |
| Youth Race (Native Hawaiian / Pacific Islander) | 0.35 | .723 | 0.12 | [-0.53, 0.76] |
| Youth Race (Other) | -0.60 | .546 | -0.05 | [-0.20, 0.11] |
| Youth Ethnicity | -3.10 | .002 | -0.06 | [-0.09, -0.02] |
| Maximum Caregiver Education | 1.22 | .221 | 0.02 | [-0.01, 0.06] |
| Questionnaire Number | -7.62 | < .001 | -0.06 | [-0.08, -0.05] |
| Family Income | -2.25 | .024 | -0.02 | [-0.04, -0.002] |
| Area Deprivation Index | -1.97 | .049 | -0.002 | [-0.003, -0.00001] |
| Increased Risk due to Job/Transit | -1.31 | .191 | -0.01 | [-0.03, 0.01] |
| Youth Age | 0.11 | .914 | 0.0001 | [-0.002, 0.002] |
| Youth Sex | 8.46 | < .001 | 0.10 | [0.08, 0.12] |
| (Maximum Caregiver Education)^2^ | 0.69 | .491 | 0.01 | [-0.01, 0.03] |
| (Questionnaire Number)^2^ | 0.05 | .964 | 0.001 | [-0.03, 0.03] |
| (Family Income)^2^ | 3.01 | .003 | 0.01 | [0.002, 0.01] |
| (Area Deprivation Index)^2^ | 1.31 | .191 | 0.00002 | [-0.00001, 0.0001] |

**Note**: The linear mixed-effects model incorporates testing the statistical significance of coefficients against a *t*-distribution. Response options to this item were on a 5-point Likert scale: 1 = Not at all, 2 = Slightly, 3 = Moderately, 4 = Very, 5 = Extremely. This item was included in all questionnaires (Q1-3). Youth Ethnicity was a categorical factor, effect coded with Hispanic/Non-Hispanic as ‑1/+1. Increase Risk due to Job/Transit was also a categorical factor, effects coded with Risk/No-Risk as -1/+1. Youth Sex was also a categorical factor, effects coded with Male/Female as -1/+1. Youth Race was also a categorical factor, in which “White” served as the reference level. Maximum Caregiver Education (i.e., highest education level between caregivers), Questionnaire Number, Area Deprivation Index, Youth Age, and Family Income were centered continuous factors. The random-effects structure included a random intercept for study site and participant ID. Random effects were restricted to be uncorrelated. Analysis included 11,898 observations across 5,649 participants. The model accounted for 61.4% of the variance in the data (*R*^2^ = .614, adjusted *R*^2^ = .613).

**Supplementary Table 20. Linear mixed-effects model output for the analysis of caregivers’ responses to, “My child seems worried about becoming ill or that others they know will become ill with coronavirus,” including the factor referring to increased household risk.**

|  | *t*(9769) | *p* | *b* | 95% CI |
| --- | --- | --- | --- | --- |
| Intercept | 32.34 | < .001 | 2.51 | [2.36, 2.66] |
| Youth Race (American Indian / Alaska Native) | 0.18 | .857 | 0.03 | [-0.33, 0.40] |
| Youth Race (Asian) | -0.04 | .969 | -0.004 | [-0.19, 0.18] |
| Youth Race (Black) | -0.20 | .839 | -0.02 | [-0.17, 0.14] |
| Youth Race (Native Hawaiian / Pacific Islander) | 0.88 | .381 | 0.27 | [-0.34, 0.88] |
| Youth Race (Other) | -0.76 | .445 | -0.06 | [-0.21, 0.09] |
| Youth Ethnicity | -5.25 | < .001 | -0.10 | [-0.13, -0.06] |
| Maximum Caregiver Education | -2.35 | .019 | -0.05 | [-0.09, -0.01] |
| Questionnaire Number | 7.86 | < .001 | 0.06 | [0.05, 0.08] |
| Family Income | -4.72 | < .001 | -0.04 | [-0.06, -0.02] |
| Area Deprivation Index | -0.48 | .633 | -0.0004 | [-0.002, 0.001] |
| Increased Risk due to Job/Transit | -1.95 | .051 | -0.02 | [-0.05, 0.0001] |
| Youth Age | -2.32 | .020 | -0.003 | [-0.01, -0.0004] |
| Youth Sex | 0.54 | .592 | 0.01 | [-0.02, 0.03] |
| (Maximum Caregiver Education)^2^ | 3.10 | .002 | 0.03 | [0.01, 0.05] |
| (Family Income)^2^ | 0.10 | .922 | 0.0002 | [-0.004, 0.01] |
| (Area Deprivation Index)^2^ | 0.80 | .427 | 0.00002 | [-0.00002, 0.0001] |

**Note**: The linear mixed-effects model incorporates testing the statistical significance of coefficients against a *t*-distribution. Response options to this item were on a 5-point Likert scale: 1 = Strongly Disagree, 2 = Disagree, 3 = Neither Disagree or Agree, 4 = Agree, 5 = Strongly Agree. This item was included in questionnaires (Q) 1 and 3. Youth Ethnicity was a categorical factor, effect coded with Hispanic/Non-Hispanic as ‑1/+1. Increase Risk due to Job/Transit was also a categorical factor, effects coded with Risk/No-Risk as -1/+1. Youth Sex was also a categorical factor, effects coded with Male/Female as -1/+1. Youth Race was also a categorical factor, in which “White” served as the reference level. Maximum Caregiver Education (i.e., highest education level between caregivers), Questionnaire Number, Area Deprivation Index, Youth Age, and Family Income were centered continuous factors. The random-effects structure included a random intercept for study site and participant ID. Random effects were restricted to be uncorrelated. Analysis included 9,786 observations across 6,040 participants. The model accounted for 52.5% of the variance in the data (*R*^2^ = .525, adjusted *R*^2^ = .525).

**Supplementary Table 21. Linear mixed-effects model output for the analysis of caregivers’ responses to, “My child seems worried about non-health related consequences of coronavirus (e.g., financial),” including the factor referring to increased household risk.**

|  | *t*(9767) | *p* | *b* | 95% CI |
| --- | --- | --- | --- | --- |
| Intercept | 32.68 | < .001 | 2.46 | [2.31, 2.61] |
| Youth Race (American Indian / Alaska Native) | 1.08 | .281 | 0.20 | [-0.16, 0.55] |
| Youth Race (Asian) | -0.61 | .543 | -0.06 | [-0.23, 0.12] |
| Youth Race (Black) | -1.99 | .047 | -0.15 | [-0.31, -0.002] |
| Youth Race (Native Hawaiian / Pacific Islander) | 0.49 | .626 | 0.15 | [-0.44, 0.73] |
| Youth Race (Other) | -0.22 | .822 | -0.02 | [-0.16, 0.13] |
| Youth Ethnicity | -1.19 | .236 | -0.02 | [-0.06, 0.01] |
| Maximum Caregiver Education | -1.49 | .137 | -0.03 | [-0.07, 0.01] |
| Questionnaire Number | 2.02 | .044 | 0.02 | [0.0005, 0.03] |
| Family Income | -8.50 | < .001 | -0.07 | [-0.09, -0.06] |
| Area Deprivation Index | -1.00 | .317 | -0.001 | [-0.002, 0.001] |
| Increased Risk due to Job/Transit | -0.52 | .602 | -0.01 | [-0.03, 0.02] |
| Youth Age | 1.08 | .282 | 0.001 | [-0.001, 0.004] |
| Youth Sex | 2.09 | .036 | 0.02 | [0.002, 0.05] |
| (Maximum Caregiver Education)^2^ | 1.37 | .171 | 0.01 | [-0.01, 0.03] |
| (Family Income)^2^ | -1.37 | .171 | -0.003 | [-0.01, 0.001] |
| (Area Deprivation Index)^2^ | 0.66 | .509 | 0.00001 | [-0.00002, 0.00005] |

**Note**: The linear mixed-effects model incorporates testing the statistical significance of coefficients against a *t*-distribution. Response options to this item were on a 5-point Likert scale: 1 = Strongly Disagree, 2 = Disagree, 3 = Neither Disagree or Agree, 4 = Agree, 5 = Strongly Agree. This item was included in questionnaires (Q) 1 and 3. Youth Ethnicity was a categorical factor, effect coded with Hispanic/Non-Hispanic as ‑1/+1. Increase Risk due to Job/Transit was also a categorical factor, effects coded with Risk/No-Risk as -1/+1. Youth Sex was also a categorical factor, effects coded with Male/Female as -1/+1. Youth Race was also a categorical factor, in which “White” served as the reference level. Maximum Caregiver Education (i.e., highest education level between caregivers), Questionnaire Number, Area Deprivation Index, Youth Age, and Family Income were centered continuous factors. The random-effects structure included a random intercept for study site and participant ID. Random effects were restricted to be uncorrelated. Analysis included 9,784 observations across 6,038 participants. The model accounted for 45.6% of the variance in the data (*R*^2^ = .456, adjusted *R*^2^ = .455).

**Supplementary Table 22. Linear mixed-effects model output for the analysis of caregivers’ response to, “Please indicate how much you talked about the following with your child in the LAST WEEK - The importance of hand washing for preventing the spread of germs.”**

|  | *t*(15063) | *p* | *b* | 95% CI |
| --- | --- | --- | --- | --- |
| Intercept | 57.25 | < .001 | 4.11 | [3.97, 4.25] |
| Caregiver Race (American Indian / Alaska Native) | -1.33 | .183 | -0.22 | [-0.54, 0.10] |
| Caregiver Race (Asian) | -0.10 | .922 | -0.01 | [-0.17, 0.15] |
| Caregiver Race (Black) | 4.09 | < .001 | 0.30 | [0.16, 0.44] |
| Caregiver Race (Native Hawaiian / Pacific Islander) | 1.43 | .151 | 0.40 | [-0.15, 0.95] |
| Caregiver Race (Other) | -1.90 | .057 | -0.14 | [-0.28, 0.00] |
| Caregiver Ethnicity | -10.51 | < .001 | -0.21 | [-0.25, -0.17] |
| Maximum Caregiver Education | -5.56 | < .001 | -0.10 | [-0.14, -0.07] |
| Questionnaire Number | -13.71 | < .001 | -0.10 | [-0.12, -0.09] |
| Family Income | -3.12 | .002 | -0.02 | [-0.04, -0.01] |
| Area Deprivation Index | -0.72 | .469 | -0.001 | [-0.002, 0.001] |
| Youth Age | -4.94 | < .001 | -0.01 | [-0.01, -0.003] |
| Youth Sex | -1.66 | .097 | -0.02 | [-0.04, 0.003] |
| (Maximum Caregiver Education)^2^ | -0.17 | .864 | -0.002 | [-0.02, 0.02] |
| (Questionnaire Number)^2^ | 2.66 | .008 | 0.03 | [0.01, 0.06] |
| (Family Income)^2^ | 0.64 | .523 | 0.001 | [-0.003, 0.01] |
| (Area Deprivation Index)^2^ | 1.30 | .193 | 0.00002 | [-0.00001, 0.0001] |

**Note**: The linear mixed-effects model incorporates testing the statistical significance of coefficients against a *t*-distribution. Response options to this item were on a 5-point Likert scale: 1 = Never, 2 = Rarely, 3 = Occasionally, 4 = Frequently, 5 = Very Frequently. This item was included in questionnaires (Q) 1, 2, and 3. Caregiver Ethnicity was a categorical factor, effect coded with Hispanic/Non-Hispanic as ‑1/+1. Youth Sex was also a categorical factor, effects coded with Male/Female as -1/+1. Caregiver Race was also a categorical factor, in which “White” served as the reference level. Maximum Caregiver Education (i.e., highest education level between caregivers), Questionnaire Number, Area Deprivation Index, Youth Age, and Family Income were centered continuous factors. The random-effects structure included a random intercept for study site and participant ID. Random effects were restricted to be uncorrelated. Analysis included 15,080 observations across 6,590 participants. The model accounted for 65.2% of the variance in the data (*R*^2^ = .652, adjusted *R*^2^ = .651).

**Supplementary Table 23. Linear mixed-effects model output for the analysis of caregivers’ response to, “Please indicate how much you talked about the following with your child in the LAST WEEK - The importance of social distancing.”**

|  | *t*(15062) | *p* | *b* | 95% CI |
| --- | --- | --- | --- | --- |
| Intercept | 57.42 | < .001 | 4.00 | [3.86, 4.13] |
| Caregiver Race (American Indian / Alaska Native) | -2.22 | .026 | -0.35 | [-0.66, -0.04] |
| Caregiver Race (Asian) | -1.63 | .104 | -0.13 | [-0.28, 0.03] |
| Caregiver Race (Black) | 3.19 | .001 | 0.22 | [0.09, 0.36] |
| Caregiver Race (Native Hawaiian / Pacific Islander) | 2.46 | .014 | 0.66 | [0.14, 1.19] |
| Caregiver Race (Other) | -1.60 | .110 | -0.11 | [-0.25, 0.03] |
| Caregiver Ethnicity | -10.45 | < .001 | -0.21 | [-0.24, -0.17] |
| Maximum Caregiver Education | -4.27 | < .001 | -0.08 | [-0.11, -0.04] |
| Questionnaire Number | -10.20 | < .001 | -0.07 | [-0.09, -0.06] |
| Family Income | -5.03 | < .001 | -0.04 | [-0.05, -0.02] |
| Area Deprivation Index | -2.81 | .005 | -0.002 | [-0.003, -0.001] |
| Youth Age | -2.73 | .006 | -0.003 | [-0.01, -0.001] |
| Youth Sex | 1.81 | .070 | 0.02 | [-0.002, 0.04] |
| (Maximum Caregiver Education)^2^ | 3.04 | .002 | 0.03 | [0.01, 0.05] |
| (Questionnaire Number)^2^ | 1.50 | .134 | 0.02 | [-0.01, 0.04] |
| (Family Income)^2^ | 3.12 | .002 | 0.01 | [0.002, 0.01] |
| (Area Deprivation Index)^2^ | 2.50 | .012 | 0.00004 | [0.00001, 0.0001] |

**Note**: The linear mixed-effects model incorporates testing the statistical significance of coefficients against a *t*-distribution. Response options to this item were on a 5-point Likert scale: 1 = Never, 2 = Rarely, 3 = Occasionally, 4 = Frequently, 5 = Very Frequently. This item was included in questionnaires (Q) 1, 2, and 3. Caregiver Ethnicity was a categorical factor, effect coded with Hispanic/Non-Hispanic as ‑1/+1. Youth Sex was also a categorical factor, effects coded with Male/Female as -1/+1. Caregiver Race was also a categorical factor, in which “White” served as the reference level. Maximum Caregiver Education (i.e., highest education level between caregivers), Questionnaire Number, Area Deprivation Index, Youth Age, and Family Income were centered continuous factors. The random-effects structure included a random intercept for study site and participant ID. Random effects were restricted to be uncorrelated. Analysis included 15,079 observations across 6,590 participants. The model accounted for 64.4% of the variance in the data (*R*^2^ = .644, adjusted *R*^2^ = .644).

**Supplementary Table 24. Linear mixed-effects model output for the analysis of caregivers’ response to, “Please indicate how much you talked about the following with your child in the LAST WEEK - Cancellation of school and other events.”**

|  | *t*(15061) | *p* | *b* | 95% CI |
| --- | --- | --- | --- | --- |
| Intercept | 48.18 | < .001 | 3.37 | [3.23, 3.51] |
| Caregiver Race (American Indian / Alaska Native) | -1.95 | .051 | -0.31 | [-0.63, 0.002] |
| Caregiver Race (Asian) | -1.97 | .049 | -0.15 | [-0.31, -0.001] |
| Caregiver Race (Black) | 2.99 | .003 | 0.21 | [0.07, 0.35] |
| Caregiver Race (Native Hawaiian / Pacific Islander) | 1.82 | .070 | 0.50 | [-0.04, 1.03] |
| Caregiver Race (Other) | -0.64 | .523 | -0.05 | [-0.19, 0.09] |
| Caregiver Ethnicity | -9.24 | < .001 | -0.18 | [-0.22, -0.14] |
| Maximum Caregiver Education | -6.33 | < .001 | -0.11 | [-0.15, -0.08] |
| Questionnaire Number | 0.03 | .975 | 0.0003 | [-0.02, 0.02] |
| Family Income | -5.27 | < .001 | -0.04 | [-0.06, -0.03] |
| Area Deprivation Index | -0.13 | .900 | -0.0001 | [-0.002, 0.001] |
| Youth Age | -1.15 | .250 | -0.001 | [-0.003, 0.001] |
| Youth Sex | 3.63 | < .001 | 0.04 | [0.02, 0.06] |
| (Maximum Caregiver Education)^2^ | 4.23 | < .001 | 0.04 | [0.02, 0.06] |
| (Questionnaire Number)^2^ | 20.55 | < .001 | 0.30 | [0.27, 0.33] |
| (Family Income)^2^ | 3.69 | < .001 | 0.01 | [0.004, 0.01] |
| (Area Deprivation Index)^2^ | 1.85 | .064 | 0.00003 | [-0.000002, 0.0001] |

**Note**: The linear mixed-effects model incorporates testing the statistical significance of coefficients against a *t*-distribution. Response options to this item were on a 5-point Likert scale: 1 = Never, 2 = Rarely, 3 = Occasionally, 4 = Frequently, 5 = Very Frequently. This item was included in questionnaires (Q) 1, 2, and 3. Caregiver Ethnicity was a categorical factor, effect coded with Hispanic/Non-Hispanic as ‑1/+1. Youth Sex was also a categorical factor, effects coded with Male/Female as -1/+1. Caregiver Race was also a categorical factor, in which “White” served as the reference level. Maximum Caregiver Education (i.e., highest education level between caregivers), Questionnaire Number, Area Deprivation Index, Youth Age, and Family Income were centered continuous factors. The random-effects structure included a random intercept for study site and participant ID. Random effects were restricted to be uncorrelated. Analysis included 15,078 observations across 6,590 participants. The model accounted for 43.8% of the variance in the data (*R*^2^ = .438, adjusted *R*^2^ = .437).

**Supplementary Table 25. Linear mixed-effects model output for the analysis of caregivers’ response to, “Please indicate how much you talked about the following with your child in the LAST WEEK - Avoiding visiting friends or family.”**

|  | *t*(15061) | *p* | *b* | 95% CI |
| --- | --- | --- | --- | --- |
| Intercept | 48.81 | < .001 | 3.52 | [3.38, 3.66] |
| Caregiver Race (American Indian / Alaska Native) | -2.12 | .034 | -0.35 | [-0.67, -0.03] |
| Caregiver Race (Asian) | -1.60 | .110 | -0.13 | [-0.29, 0.03] |
| Caregiver Race (Black) | 1.72 | .086 | 0.13 | [-0.02, 0.27] |
| Caregiver Race (Native Hawaiian / Pacific Islander) | 2.92 | .004 | 0.82 | [0.27, 1.37] |
| Caregiver Race (Other) | -2.15 | .032 | -0.16 | [-0.30, -0.01] |
| Caregiver Ethnicity | -11.34 | < .001 | -0.23 | [-0.27, -0.19] |
| Maximum Caregiver Education | -6.23 | < .001 | -0.12 | [-0.15, -0.08] |
| Questionnaire Number | -19.28 | < .001 | -0.16 | [-0.17, -0.14] |
| Family Income | -7.11 | < .001 | -0.06 | [-0.07, -0.04] |
| Area Deprivation Index | -3.06 | .002 | -0.002 | [-0.004, -0.001] |
| Youth Age | -2.76 | .006 | -0.003 | [-0.01, -0.001] |
| Youth Sex | 2.78 | .005 | 0.03 | [0.01, 0.05] |
| (Maximum Caregiver Education)^2^ | 4.81 | < .001 | 0.05 | [0.03, 0.07] |
| (Questionnaire Number)^2^ | 5.22 | < .001 | 0.07 | [0.04, 0.10] |
| (Family Income)^2^ | 2.20 | .028 | 0.005 | [0.001, 0.01] |
| (Area Deprivation Index)^2^ | 3.26 | .001 | 0.0001 | [0.00002, 0.0001] |

**Note**: The linear mixed-effects model incorporates testing the statistical significance of coefficients against a *t*-distribution. Response options to this item were on a 5-point Likert scale: 1 = Never, 2 = Rarely, 3 = Occasionally, 4 = Frequently, 5 = Very Frequently. This item was included in questionnaires (Q) 1, 2, and 3. Caregiver Ethnicity was a categorical factor, effect coded with Hispanic/Non-Hispanic as ‑1/+1. Youth Sex was also a categorical factor, effects coded with Male/Female as -1/+1. Caregiver Race was also a categorical factor, in which “White” served as the reference level. Maximum Caregiver Education (i.e., highest education level between caregivers), Questionnaire Number, Area Deprivation Index, Youth Age, and Family Income were centered continuous factors. The random-effects structure included a random intercept for study site and participant ID. Random effects were restricted to be uncorrelated. Analysis included 15,078 observations across 6,590 participants. The model accounted for 55.7% of the variance in the data (*R*^2^ = .557, adjusted *R*^2^ = .556).

**Supplementary Table 26. Linear mixed-effects model output for the analysis of caregivers’ response to, “Please indicate how much you talked about the following with your child in the LAST WEEK - The symptoms of coronavirus.”**

|  | *t*(15057) | *p* | *b* | 95% CI |
| --- | --- | --- | --- | --- |
| Intercept | 42.95 | < .001 | 3.21 | [3.06, 3.35] |
| Caregiver Race (American Indian / Alaska Native) | -1.86 | .063 | -0.32 | [-0.66, 0.02] |
| Caregiver Race (Asian) | 1.00 | .320 | 0.08 | [-0.08, 0.25] |
| Caregiver Race (Black) | 3.96 | < .001 | 0.30 | [0.15, 0.46] |
| Caregiver Race (Native Hawaiian / Pacific Islander) | 1.15 | .250 | 0.34 | [-0.24, 0.92] |
| Caregiver Race (Other) | -1.05 | .293 | -0.08 | [-0.23, 0.07] |
| Caregiver Ethnicity | -13.19 | < .001 | -0.28 | [-0.32, -0.24] |
| Maximum Caregiver Education | -5.76 | < .001 | -0.11 | [-0.15, -0.07] |
| Questionnaire Number | -1.75 | .080 | -0.01 | [-0.03, 0.002] |
| Family Income | -6.08 | < .001 | -0.05 | [-0.07, -0.03] |
| Area Deprivation Index | 1.71 | .087 | 0.001 | [-0.0002, 0.003] |
| Youth Age | 1.04 | .298 | 0.001 | [-0.001, 0.004] |
| Youth Sex | 1.55 | .122 | 0.02 | [-0.01, 0.04] |
| (Maximum Caregiver Education)^2^ | -0.82 | .414 | -0.01 | [-0.03, 0.01] |
| (Questionnaire Number)^2^ | 0.53 | .598 | 0.01 | [-0.02, 0.03] |
| (Family Income)^2^ | 0.92 | .359 | 0.002 | [-0.002, 0.01] |
| (Area Deprivation Index)^2^ | 0.48 | .633 | 0.00001 | [-0.00003, 0.00005] |

**Note**: The linear mixed-effects model incorporates testing the statistical significance of coefficients against a *t*-distribution. Response options to this item were on a 5-point Likert scale: 1 = Never, 2 = Rarely, 3 = Occasionally, 4 = Frequently, 5 = Very Frequently. This item was included in questionnaires (Q) 1, 2, and 3. Caregiver Ethnicity was a categorical factor, effect coded with Hispanic/Non-Hispanic as ‑1/+1. Youth Sex was also a categorical factor, effects coded with Male/Female as -1/+1. Caregiver Race was also a categorical factor, in which “White” served as the reference level. Maximum Caregiver Education (i.e., highest education level between caregivers), Questionnaire Number, Area Deprivation Index, Youth Age, and Family Income were centered continuous factors. The random-effects structure included a random intercept for study site and participant ID. Random effects were restricted to be uncorrelated. Analysis included 15,074 observations across 6,589 participants. The model accounted for 59.2% of the variance in the data (*R*^2^ = .592, adjusted *R*^2^ = .592).

**Supplementary Table 27. Linear mixed-effects model output for the analysis of caregivers’ response to, “Please indicate how much you talked about the following with your child in the LAST WEEK - Protecting the elderly or other vulnerable people.”**

|  | *t*(15061) | *p* | *b* | 95% CI |
| --- | --- | --- | --- | --- |
| Intercept | 46.91 | < .001 | 3.67 | [3.52, 3.82] |
| Caregiver Race (American Indian / Alaska Native) | -1.72 | .086 | -0.31 | [-0.66, 0.04] |
| Caregiver Race (Asian) | -1.33 | .184 | -0.12 | [-0.29, 0.06] |
| Caregiver Race (Black) | 0.01 | .994 | 0.001 | [-0.16, 0.16] |
| Caregiver Race (Native Hawaiian / Pacific Islander) | 2.89 | .004 | 0.89 | [0.28, 1.49] |
| Caregiver Race (Other) | -1.49 | .136 | -0.12 | [-0.28, 0.04] |
| Caregiver Ethnicity | -10.43 | < .001 | -0.23 | [-0.27, -0.19] |
| Maximum Caregiver Education | -7.00 | < .001 | -0.14 | [-0.18, -0.10] |
| Questionnaire Number | -8.29 | < .001 | -0.07 | [-0.08, -0.05] |
| Family Income | -4.55 | < .001 | -0.04 | [-0.06, -0.02] |
| Area Deprivation Index | -0.72 | .475 | -0.001 | [-0.002, 0.001] |
| Youth Age | -0.43 | .666 | -0.001 | [-0.003, 0.002] |
| Youth Sex | 0.89 | .375 | 0.01 | [-0.01, 0.04] |
| (Maximum Caregiver Education)^2^ | 2.37 | .018 | 0.03 | [0.004, 0.05] |
| (Questionnaire Number)^2^ | -1.20 | .232 | -0.02 | [-0.04, 0.01] |
| (Family Income)^2^ | 2.58 | .010 | 0.01 | [0.001, 0.01] |
| (Area Deprivation Index)^2^ | 1.84 | .065 | 0.00004 | [-0.000002, 0.0001] |

**Note**: The linear mixed-effects model incorporates testing the statistical significance of coefficients against a *t*-distribution. Response options to this item were on a 5-point Likert scale: 1 = Never, 2 = Rarely, 3 = Occasionally, 4 = Frequently, 5 = Very Frequently. This item was included in questionnaires (Q) 1, 2, and 3. Caregiver Ethnicity was a categorical factor, effect coded with Hispanic/Non-Hispanic as ‑1/+1. Youth Sex was also a categorical factor, effects coded with Male/Female as -1/+1. Caregiver Race was also a categorical factor, in which “White” served as the reference level. Maximum Caregiver Education (i.e., highest education level between caregivers), Questionnaire Number, Area Deprivation Index, Youth Age, and Family Income were centered continuous factors. The random-effects structure included a random intercept for study site and participant ID. Random effects were restricted to be uncorrelated. Analysis included 15,078 observations across 6,590 participants. The model accounted for 62.2% of the variance in the data (*R*^2^ = .622, adjusted *R*^2^ = .622).

**Supplementary Table 28. Linear mixed-effects model output for the analysis of caregivers’ response to, “Please indicate how much you talked about the following with your child in the LAST WEEK - The importance of wearing a mask.”**

|  | *t*(4804) | *p* | *b* | 95% CI |
| --- | --- | --- | --- | --- |
| Intercept | 47.43 | < .001 | 4.15 | [3.98, 4.32] |
| Caregiver Race (American Indian / Alaska Native) | -1.37 | .171 | -0.30 | [-0.73, 0.13] |
| Caregiver Race (Asian) | -1.83 | .068 | -0.18 | [-0.38, 0.01] |
| Caregiver Race (Black) | 2.35 | .019 | 0.22 | [0.04, 0.40] |
| Caregiver Race (Native Hawaiian / Pacific Islander) | 1.88 | .060 | 0.62 | [-0.03, 1.26] |
| Caregiver Race (Other) | -0.45 | .656 | -0.04 | [-0.22, 0.14] |
| Caregiver Ethnicity | -7.19 | < .001 | -0.20 | [-0.25, -0.14] |
| Maximum Caregiver Education | 0.04 | .971 | 0.001 | [-0.05, 0.05] |
| Family Income | -3.34 | .001 | -0.04 | [-0.06, -0.01] |
| Area Deprivation Index | -2.34 | .019 | -0.002 | [-0.004, -0.0004] |
| Youth Age | -2.18 | .029 | -0.003 | [-0.01, -0.0003] |
| Youth Sex | 0.87 | .386 | 0.01 | [-0.02, 0.04] |
| (Maximum Caregiver Education)^2^ | 0.45 | .653 | 0.01 | [-0.02, 0.03] |
| (Family Income)^2^ | 4.22 | < .001 | 0.01 | [0.01, 0.02] |
| (Area Deprivation Index)^2^ | 2.41 | .016 | 0.0001 | [0.00001, 0.0001] |

**Note**: The linear mixed-effects model incorporates testing the statistical significance of coefficients against a *t*-distribution. Response options to this item were on a 5-point Likert scale: 1 = Never, 2 = Rarely, 3 = Occasionally, 4 = Frequently, 5 = Very Frequently. This item was included in questionnaire (Q) 2. Caregiver Ethnicity was a categorical factor, effect coded with Hispanic/Non-Hispanic as ‑1/+1. Youth Sex was also a categorical factor, effects coded with Male/Female as -1/+1. Caregiver Race was also a categorical factor, in which “White” served as the reference level. Maximum Caregiver Education (i.e., highest education level between caregivers), Area Deprivation Index, Youth Age, and Family Income were centered continuous factors. The random-effects structure included a random intercept for study site. Analysis included 4,819 data points. The model accounted for 6.5% of the variance in the data (*R*^2^ = .065, adjusted *R*^2^ = .063).

**Supplementary Table 29. Linear mixed-effects model output for the analysis of caregivers’ response to, “I have told my child that everything will be okay.”**

|  | *t*(9842) | *p* | *b* | 95% CI |
| --- | --- | --- | --- | --- |
| Intercept | 58.48 | < .001 | 4.02 | [3.88, 4.15] |
| Caregiver Race (American Indian / Alaska Native) | -0.43 | .671 | -0.06 | [-0.36, 0.23] |
| Caregiver Race (Asian) | -1.29 | .198 | -0.10 | [-0.24, 0.05] |
| Caregiver Race (Black) | 1.05 | .292 | 0.07 | [-0.06, 0.21] |
| Caregiver Race (Native Hawaiian / Pacific Islander) | 0.40 | .689 | 0.11 | [-0.42, 0.63] |
| Caregiver Race (Other) | -0.92 | .357 | -0.06 | [-0.20, 0.07] |
| Caregiver Ethnicity | -4.97 | < .001 | -0.09 | [-0.13, -0.06] |
| Maximum Caregiver Education | -3.86 | < .001 | -0.07 | [-0.10, -0.03] |
| Questionnaire Number | -8.79 | < .001 | -0.06 | [-0.07, -0.05] |
| Family Income | -1.18 | .239 | -0.01 | [-0.02, 0.01] |
| Area Deprivation Index | 2.58 | .010 | 0.002 | [0.0004, 0.003] |
| Youth Age | -5.05 | < .001 | -0.01 | [-0.01, -0.003] |
| Youth Sex | 0.98 | .326 | 0.01 | [-0.01, 0.03] |
| (Maximum Caregiver Education)^2^ | -0.50 | .615 | -0.005 | [-0.02, 0.01] |
| (Family Income)^2^ | -0.83 | .407 | -0.002 | [-0.01, 0.002] |
| (Area Deprivation Index)^2^ | -0.63 | .527 | -0.00001 | [-0.00004, 0.00002] |

**Note**: The linear mixed-effects model incorporates testing the statistical significance of coefficients against a *t*-distribution. Response options to this item were on a 5-point Likert scale: 1 = Strongly Disagree, 2 = Disagree, 3 = Neither Disagree or Agree, 4 = Agree, 5 = Strongly Agree. In the main text, this item is referred to as *caregiver reassurance*. This item was included in questionnaires (Q) 1 and 3. Caregiver Ethnicity was a categorical factor, effect coded with Hispanic/Non-Hispanic as ‑1/+1. Youth Sex was also a categorical factor, effects coded with Male/Female as -1/+1. Caregiver Race was also a categorical factor, in which “White” served as the reference level. Maximum Caregiver Education (i.e., highest education level between caregivers), Questionnaire Number, Area Deprivation Index, Youth Age, and Family Income were centered continuous factors. The random-effects structure included a random intercept for study site and participant ID. Random effects were restricted to be uncorrelated. Analysis included 9,858 observations across 6,072 participants. The model accounted for 52.9% of the variance in the data (*R*^2^ = .529, adjusted *R*^2^ = .529).

**Supplementary Table 30. Linear mixed-effects model output for the analysis of caregivers’ response to, “I have encouraged my child not to focus on coronavirus or its impacts on people and the world.”**

|  | *t*(9839) | *p* | *b* | 95% CI |
| --- | --- | --- | --- | --- |
| Intercept | 45.26 | < .001 | 3.17 | [3.04, 3.31] |
| Caregiver Race (American Indian / Alaska Native) | 0.48 | .631 | 0.08 | [-0.24, 0.39] |
| Caregiver Race (Asian) | -1.49 | .137 | -0.12 | [-0.27, 0.04] |
| Caregiver Race (Black) | 0.19 | .847 | 0.01 | [-0.13, 0.16] |
| Caregiver Race (Native Hawaiian / Pacific Islander) | 0.60 | .548 | 0.17 | [-0.38, 0.72] |
| Caregiver Race (Other) | -1.23 | .218 | -0.09 | [-0.23, 0.05] |
| Caregiver Ethnicity | -4.67 | < .001 | -0.09 | [-0.13, -0.05] |
| Maximum Caregiver Education | -5.83 | < .001 | -0.11 | [-0.14, -0.07] |
| Questionnaire Number | -4.19 | < .001 | -0.03 | [-0.05, -0.02] |
| Family Income | -2.72 | .007 | -0.02 | [-0.04, -0.01] |
| Area Deprivation Index | 2.43 | .015 | 0.002 | [0.0003, 0.003] |
| Youth Age | -2.05 | .040 | -0.002 | [-0.004, -0.0001] |
| Youth Sex | -0.85 | .395 | -0.01 | [-0.03, 0.01] |
| (Maximum Caregiver Education)^2^ | 1.11 | .266 | 0.01 | [-0.01, 0.03] |
| (Family Income)^2^ | -3.58 | < .001 | -0.01 | [-0.01, -0.003] |
| (Area Deprivation Index)^2^ | -2.07 | .039 | -0.00004 | [-0.0001,  -0.000002] |

**Note**: The linear mixed-effects model incorporates testing the statistical significance of coefficients against a *t*-distribution. Response options to this item were on a 5-point Likert scale: 1 = Strongly Disagree, 2 = Disagree, 3 = Neither Disagree or Agree, 4 = Agree, 5 = Strongly Agree. In the main text, this item is referred to as *caregiver encouragement*. This item was included in questionnaires (Q) 1 and 3. Caregiver Ethnicity was a categorical factor, effect coded with Hispanic/Non-Hispanic as ‑1/+1. Youth Sex was also a categorical factor, effects coded with Male/Female as -1/+1. Caregiver Race was also a categorical factor, in which “White” served as the reference level. Maximum Caregiver Education (i.e., highest education level between caregivers), Questionnaire Number, Area Deprivation Index, Youth Age, and Family Income were centered continuous factors. The random-effects structure included a random intercept for study site and participant ID. Random effects were restricted to be uncorrelated. Analysis included 9,855 observations across 6,071 participants. The model accounted for 33.2% of the variance in the data (*R*^2^ = .332, adjusted *R*^2^ = .331).

**Supplementary Table 31. Linear mixed-effects model output for the analysis of caregivers’ response to, “I discussed with my child my own feelings about coronavirus and its impact on people and the world.”**

|  | *t*(9841) | *p* | *b* | 95% CI |
| --- | --- | --- | --- | --- |
| Intercept | 52.01 | < .001 | 3.61 | [3.47, 3.74] |
| Caregiver Race (American Indian / Alaska Native) | -1.76 | .079 | -0.28 | [-0.60, 0.03] |
| Caregiver Race (Asian) | -1.52 | .129 | -0.12 | [-0.28, 0.03] |
| Caregiver Race (Black) | 0.37 | .715 | 0.03 | [-0.12, 0.17] |
| Caregiver Race (Native Hawaiian / Pacific Islander) | 1.71 | .087 | 0.49 | [-0.07, 1.05] |
| Caregiver Race (Other) | -0.22 | .829 | -0.02 | [-0.16, 0.13] |
| Caregiver Ethnicity | 1.65 | .100 | 0.03 | [-0.01, 0.07] |
| Maximum Caregiver Education | 2.63 | .009 | 0.05 | [0.01, 0.08] |
| Questionnaire Number | -0.84 | .403 | -0.01 | [-0.02, 0.01] |
| Family Income | -0.39 | .698 | -0.003 | [-0.02, 0.01] |
| Area Deprivation Index | -0.21 | .836 | -0.0001 | [-0.001, 0.001] |
| Youth Age | 1.95 | .052 | 0.002 | [-0.00002, 0.004] |
| Youth Sex | 1.78 | .075 | 0.02 | [-0.002, 0.04] |
| (Maximum Caregiver Education)^2^ | -0.94 | .348 | -0.01 | [-0.03, 0.01] |
| (Family Income)^2^ | 2.50 | .013 | 0.01 | [0.001, 0.01] |
| (Area Deprivation Index)^2^ | 1.02 | .305 | 0.00002 | [-0.00002, 0.0001] |

**Note**: The linear mixed-effects model incorporates testing the statistical significance of coefficients against a *t*-distribution. Response options to this item were on a 5-point Likert scale: 1 = Strongly Disagree, 2 = Disagree, 3 = Neither Disagree or Agree, 4 = Agree, 5 = Strongly Agree. This item was included in questionnaires (Q) 1 and 3. Caregiver Ethnicity was a categorical factor, effect coded with Hispanic/Non-Hispanic as ‑1/+1. Youth Sex was also a categorical factor, effects coded with Male/Female as -1/+1. Caregiver Race was also a categorical factor, in which “White” served as the reference level. Maximum Caregiver Education (i.e., highest education level between caregivers), Questionnaire Number, Area Deprivation Index, Youth Age, and Family Income were centered continuous factors. The random-effects structure included a random intercept for study site and participant ID. Random effects were restricted to be uncorrelated. Analysis included 9,857 observations across 6,071 participants. The model accounted for 42.4% of the variance in the data (*R*^2^ = .424, adjusted *R*^2^ = .423).

**Supplementary Table 32. Linear mixed-effects model output for the analysis of caregivers’ response to, “I have avoided talking to my child about coronavirus.”**

|  | *t*(9842) | *p* | *b* | 95% CI |
| --- | --- | --- | --- | --- |
| Intercept | 36.85 | < .001 | 2.05 | [1.94, 2.16] |
| Caregiver Race (American Indian / Alaska Native) | 0.67 | .501 | 0.09 | [-0.17, 0.34] |
| Caregiver Race (Asian) | -0.06 | .954 | -0.004 | [-0.13, 0.12] |
| Caregiver Race (Black) | -1.58 | .115 | -0.09 | [-0.21, 0.02] |
| Caregiver Race (Native Hawaiian / Pacific Islander) | 0.96 | .336 | 0.22 | [-0.23, 0.67] |
| Caregiver Race (Other) | -1.71 | .087 | -0.10 | [-0.22, 0.01] |
| Caregiver Ethnicity | -5.50 | < .001 | -0.08 | [-0.11, -0.05] |
| Maximum Caregiver Education | -6.53 | < .001 | -0.10 | [-0.12, -0.07] |
| Questionnaire Number | 2.06 | .039 | 0.01 | [0.001, 0.03] |
| Family Income | -5.09 | < .001 | -0.03 | [-0.04, -0.02] |
| Area Deprivation Index | 0.02 | .980 | 0.00001 | [-0.001, 0.001] |
| Youth Age | -3.18 | .001 | -0.003 | [-0.004, -0.001] |
| Youth Sex | 0.15 | .878 | 0.001 | [-0.02, 0.02] |
| (Maximum Caregiver Education)^2^ | 3.28 | .001 | 0.02 | [0.01, 0.04] |
| (Family Income)^2^ | -1.67 | .095 | -0.003 | [-0.01, 0.0005] |
| (Area Deprivation Index)^2^ | 0.19 | .847 | 0.000003 | [-0.00002, 0.00003] |

**Note**: The linear mixed-effects model incorporates testing the statistical significance of coefficients against a *t*-distribution. Response options to this item were on a 5-point Likert scale: 1 = Strongly Disagree, 2 = Disagree, 3 = Neither Disagree or Agree, 4 = Agree, 5 = Strongly Agree. This item was included in questionnaires (Q) 1 and 3. Caregiver Ethnicity was a categorical factor, effect coded with Hispanic/Non-Hispanic as ‑1/+1. Youth Sex was also a categorical factor, effects coded with Male/Female as -1/+1. Caregiver Race was also a categorical factor, in which “White” served as the reference level. Maximum Caregiver Education (i.e., highest education level between caregivers), Questionnaire Number, Area Deprivation Index, Youth Age, and Family Income were centered continuous factors. The random-effects structure included a random intercept for study site and participant ID. Random effects were restricted to be uncorrelated. Analysis included 9,858 observations across 6,072 participants. The model accounted for 30.9% of the variance in the data (*R*^2^ = .309, adjusted *R*^2^ = .308).

**Supplementary Table 33. Linear mixed-effects model output for the analysis of caregivers’ response to, “I have expressed concern to my child that they might not be fully safe from coronavirus.”**

|  | *t*(9841) | *p* | *b* | 95% CI |
| --- | --- | --- | --- | --- |
| Intercept | 38.31 | < .001 | 3.19 | [3.03, 3.35] |
| Caregiver Race (American Indian / Alaska Native) | -0.53 | .593 | -0.10 | [-0.48, 0.28] |
| Caregiver Race (Asian) | -0.62 | .538 | -0.06 | [-0.24, 0.13] |
| Caregiver Race (Black) | 0.12 | .905 | 0.01 | [-0.16, 0.18] |
| Caregiver Race (Native Hawaiian / Pacific Islander) | 2.26 | .024 | 0.77 | [0.10, 1.44] |
| Caregiver Race (Other) | -2.73 | .006 | -0.24 | [-0.41, -0.07] |
| Caregiver Ethnicity | -1.85 | .065 | -0.04 | [-0.09, 0.003] |
| Maximum Caregiver Education | -2.03 | .042 | -0.04 | [-0.09, -0.002] |
| Questionnaire Number | 10.75 | < .001 | 0.10 | [0.08, 0.12] |
| Family Income | -4.40 | < .001 | -0.04 | [-0.06, -0.02] |
| Area Deprivation Index | -0.08 | .935 | -0.0001 | [-0.002, 0.002] |
| Youth Age | 3.06 | .002 | 0.004 | [0.001, 0.01] |
| Youth Sex | 1.63 | .104 | 0.02 | [-0.004, 0.05] |
| (Maximum Caregiver Education)^2^ | 1.37 | .170 | 0.02 | [-0.01, 0.04] |
| (Family Income)^2^ | 1.57 | .117 | 0.004 | [-0.001, 0.01] |
| (Area Deprivation Index)^2^ | 1.04 | .300 | 0.00002 | [-0.00002, 0.0001] |

**Note**: The linear mixed-effects model incorporates testing the statistical significance of coefficients against a *t*-distribution. Response options to this item were on a 5-point Likert scale: 1 = Strongly Disagree, 2 = Disagree, 3 = Neither Disagree or Agree, 4 = Agree, 5 = Strongly Agree. This item was included in questionnaires (Q) 1 and 3. Caregiver Ethnicity was a categorical factor, effect coded with Hispanic/Non-Hispanic as ‑1/+1. Youth Sex was also a categorical factor, effects coded with Male/Female as -1/+1. Caregiver Race was also a categorical factor, in which “White” served as the reference level. Maximum Caregiver Education (i.e., highest education level between caregivers), Questionnaire Number, Area Deprivation Index, Youth Age, and Family Income were centered continuous factors. The random-effects structure included a random intercept for study site and participant ID. Random effects were restricted to be uncorrelated. Analysis included 9,857 observations across 6,071 participants. The model accounted for 44.3% of the variance in the data (*R*^2^ = .443, adjusted *R*^2^ = .442).

**Supplementary Table 34. Linear mixed-effects model output for the analysis of caregivers’ response to, “I have prepared my child for our lives to change significantly.”**

|  | *t*(9842) | *p* | *b* | 95% CI |
| --- | --- | --- | --- | --- |
| Intercept | 50.45 | < .001 | 3.64 | [3.50, 3.78] |
| Caregiver Race (American Indian / Alaska Native) | -2.22 | .026 | -0.36 | [-0.68, -0.04] |
| Caregiver Race (Asian) | -0.55 | .579 | -0.04 | [-0.20, 0.11] |
| Caregiver Race (Black) | 1.45 | .147 | 0.11 | [-0.04, 0.25] |
| Caregiver Race (Native Hawaiian / Pacific Islander) | 1.66 | .097 | 0.48 | [-0.09, 1.04] |
| Caregiver Race (Other) | -0.47 | .637 | -0.03 | [-0.18, 0.11] |
| Caregiver Ethnicity | -3.35 | .001 | -0.07 | [-0.10, -0.03] |
| Maximum Caregiver Education | -0.71 | .476 | -0.01 | [-0.05, 0.02] |
| Questionnaire Number | -1.90 | .058 | -0.01 | [-0.03, 0.0005] |
| Family Income | -4.28 | < .001 | -0.03 | [-0.05, -0.02] |
| Area Deprivation Index | -0.68 | .497 | -0.0005 | [-0.002, 0.001] |
| Youth Age | -2.66 | .008 | -0.003 | [-0.01, -0.001] |
| Youth Sex | 1.68 | .092 | 0.02 | [-0.003, 0.04] |
| (Maximum Caregiver Education)^2^ | 1.79 | .073 | 0.02 | [-0.002, 0.04] |
| (Family Income)^2^ | 0.21 | .837 | 0.0004 | [-0.004, 0.01] |
| (Area Deprivation Index)^2^ | 0.82 | .412 | 0.00001 | [-0.00002, 0.00005] |

**Note**: The linear mixed-effects model incorporates testing the statistical significance of coefficients against a *t*-distribution. Response options to this item were on a 5-point Likert scale: 1 = Strongly Disagree, 2 = Disagree, 3 = Neither Disagree or Agree, 4 = Agree, 5 = Strongly Agree. This item was included in questionnaires (Q) 1 and 3. Caregiver Ethnicity was a categorical factor, effect coded with Hispanic/Non-Hispanic as ‑1/+1. Youth Sex was also a categorical factor, effects coded with Male/Female as -1/+1. Caregiver Race was also a categorical factor, in which “White” served as the reference level. Maximum Caregiver Education (i.e., highest education level between caregivers), Questionnaire Number, Area Deprivation Index, Youth Age, and Family Income were centered continuous factors. The random-effects structure included a random intercept for study site and participant ID. Random effects were restricted to be uncorrelated. Analysis included 9,858 observations across 6,072 participants. The model accounted for 40.7% of the variance in the data (*R*^2^ = .407, adjusted *R*^2^ = .406).

**Supplementary Table 35. Linear mixed-effects model output for the analysis of youths’ average responses to endorsing COVID-19 preventative behaviors.**

|  | *t*(8071) | *p* | *b* | 95% CI |
| --- | --- | --- | --- | --- |
| Intercept | 44.76 | < .001 | 2.73 | [2.61, 2.85] |
| Youth Race (American Indian / Alaska Native) | -1.81 | .070 | -0.23 | [-0.48, 0.02] |
| Youth Race (Asian) | -1.25 | .212 | -0.09 | [-0.22, 0.05] |
| Youth Race (Black) | 2.12 | .034 | 0.13 | [0.01, 0.25] |
| Youth Race (Native Hawaiian / Pacific Islander) | 1.86 | .063 | 0.47 | [-0.03, 0.96] |
| Youth Race (Other) | -1.59 | .113 | -0.09 | [-0.21, 0.02] |
| Youth Ethnicity | -5.19 | < .001 | -0.07 | [-0.09, -0.04] |
| Maximum Caregiver Education | -2.68 | .007 | -0.04 | [-0.06, -0.01] |
| Questionnaire Number | 4.55 | < .001 | 0.02 | [0.01, 0.03] |
| Family Income | -4.05 | < .001 | -0.02 | [-0.04, -0.01] |
| Area Deprivation Index | -2.63 | .009 | -0.001 | [-0.003, -0.0004] |
| Youth Age | -0.11 | .913 | -0.0001 | [-0.002, 0.002] |
| Youth Sex | 10.04 | < .001 | 0.08 | [0.07, 0.10] |
| (Maximum Caregiver Education)^2^ | 2.26 | .024 | 0.02 | [0.002, 0.03] |
| (Family Income)^2^ | 3.48 | .001 | 0.01 | [0.002, 0.01] |
| (Area Deprivation Index)^2^ | 3.41 | .001 | 0.00005 | [0.00002, 0.0001] |

**Note**: The linear mixed-effects model incorporates testing the statistical significance of coefficients against a *t*-distribution. The averaged items were, “I wear a mask over my face or protective gear (e.g. gloves, things to cover my clothes),” “I stay away from people (other than those who live in my house),” “I stay away from people inside my house (e.g., stay in another room or a certain distance away),” “I use Purell/other hand sanitizer,” “I wash my hands at times other than just after I use the bathroom or before eating,” “I use Clorox/cleaners to wipe down surfaces,” “I avoid touching things (e.g., phone, doorknobs),” and “I avoid touching people (e.g., hugging, shaking hands).” Response options for each item were on a 4-point Likert scale: 1 = I have not done this in the last week, 2 = I did this some of the time last week, 3 = I did this most of the time last week, 4 = I did this all the time last week. This item was included in questionnaires (Q) 1 and 3. Youth Ethnicity was a categorical factor, effect coded with Hispanic/Non-Hispanic as ‑1/+1. Youth Sex was also a categorical factor, effects coded with Male/Female as -1/+1. Youth Race was also a categorical factor, in which “White” served as the reference level. Maximum Caregiver Education (i.e., highest education level between caregivers), Questionnaire Number, Area Deprivation Index, Youth Age, and Family Income were centered continuous factors. The random-effects structure included a random intercept for study site and participant ID. Random effects were restricted to be uncorrelated. Analysis included 8,087 observations across 5,246 participants. The model accounted for 67.4% of the variance in the data (*R*^2^ = .674, adjusted *R*^2^ = .673).
